# MicroRNA-200 Loaded Lipid Nanoparticles Promote Intestinal Epithelium Regeneration in Canonical MicroRNA-Deficient Mice

**DOI:** 10.1021/acsnano.3c08030

**Published:** 2023-11-08

**Authors:** Xiyang Wei, Shicheng Yu, Tinghong Zhang, Liansheng Liu, Xu Wang, Xiaodan Wang, Yun-Shen Chan, Yangming Wang, Shu Meng, Ye-Guang Chen

**Affiliations:** † Guangzhou Institutes of Biomedicine and Health, Chinese Academy of Sciences, Guangzhou 510530, China; ‡ Guangzhou National Laboratory, Guangzhou 510005, China; § The State Key Laboratory of Membrane Biology, Tsinghua-Peking Center for Life Sciences, School of Life Sciences, Tsinghua University, Beijing 100084, China; ∥ Institute of Molecular Medicine, College of Future Technology, Peking University, Beijing 100871, China; ⊥ School of Basic Medicine, Jiangxi Medical College, Nanchang University, Nanchang 330031, China

**Keywords:** intestinal regeneration, lipid nanoparticle, microRNA, intestinal stem cell, oral delivery

## Abstract

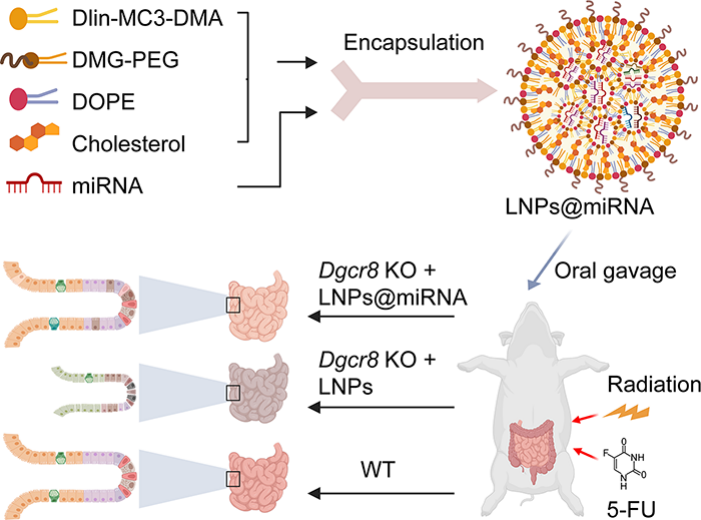

Intestinal epithelium undergoes regeneration after injuries, and the disruption of this process can lead to inflammatory bowel disease and tumorigenesis. Intestinal stem cells (ISCs) residing in the crypts are crucial for maintaining the intestinal epithelium’s homeostasis and promoting regeneration upon injury. However, the precise role of DGCR8, a critical component in microRNA (miRNA) biogenesis, in intestinal regeneration remains poorly understood. In this study, we provide compelling evidence demonstrating the indispensable role of epithelial miRNAs in the regeneration of the intestine in mice subjected to 5-FU or irradiation-induced injury. Through a comprehensive pooled screen of miRNA function in *Dgcr8*-deficient organoids, we observe that the loss of the miR-200 family leads to the hyperactivation of the p53 pathway, thereby reducing ISCs and impairing epithelial regeneration. Notably, downregulation of the miR-200 family and hyperactivation of the p53 pathway are verified in colonic tissues from patients with active ulcerative colitis (UC). Most importantly, the transient supply of miR-200 through the oral delivery of lipid nanoparticles (LNPs) carrying miR-200 restores ISCs and promotes intestinal regeneration in mice following acute injury. Our study implies the miR-200/p53 pathway as a promising therapeutic target for active UC patients with diminished levels of the miR-200 family. Furthermore, our findings suggest that the clinical application of LNP-miRNAs could enhance the efficacy, safety, and acceptability of existing therapeutic modalities for intestinal diseases.

## Introduction

The intestinal epithelium has an ability to regenerate rapidly in response to various types of damage.^1^ This regenerative capacity is regulated by multiple stem and progenitor populations found in the intestinal crypts.^2,3^ ISCs are vital for maintaining the intestinal epithelium’s homeostasis by generating all cell types along the crypt-villi axis.^4,5^ Exposure to irradiation and chemotherapeutic agents can deplete actively proliferating ISCs and transit-amplifying (TA) cells in the intestinal epithelium.^6^ Lgr5 marks the ISCs in the intestine, and these Lgr5^+^ ISCs are essential for intestinal regeneration following acute injury.^7,8^

MiRNAs are short, noncoding RNAs approximately 22 nucleotides in length. They bind to the 3′ untranslated regions (3′-UTRs) of target mRNAs, leading to post-transcriptional gene silencing.^9,10^ DGCR8 (DiGeorge syndrome critical region 8) is a vital component in the biogenesis of canonical miRNAs.^9,11^ Increasing evidence suggests that miRNAs are involved in the complex regulatory networks that control intestinal epithelial cells during development, adult tissue renewal, and disease progression.^12^ For instance, miR-34a directly targets Numb, influencing asymmetric cell division of ISCs and suppressing stem cell proliferation.^13^ miR-31 promotes ISC proliferation and epithelial regeneration following injury by regulating Wnt and Hippo signaling.^14,15^ Additionally, miR-802 targets Tmed9 and regulates intestinal epithelial cell proliferation and Paneth cell function.^16^ However, the comprehensive role of canonical miRNAs in intestinal regeneration remains largely unknown, necessitating further exploration. Additionally, the clinical application of miRNAs in therapeutic approaches for intestinal diseases requires extensive investigation.

In the pursuit of targeted therapies for specific cells, there is ongoing development to ensure their functionality within the desired target cells while minimizing the interaction with the immune system. Lipid nanoparticles (LNPs), as a crucial class of drug delivery systems, have been shown to successfully deliver mRNAs and siRNA to various types of cells, including hepatocytes, pulmonary and cardiovascular endothelial cells, bone marrow, splenic endothelial cells, and lung epithelial cells.^17^ For instance, the oral administration of LNPs-IL-22 mRNA in a mouse model of acute colitis demonstrated a notable enhancement in the protein expression of IL-22 in the colonic mucosa, thereby facilitating the recovery process.^18^ However, the impact of LNPs-miRNAs on intestinal regeneration remains ambiguous.

In this study, we present compelling evidence illustrating the crucial role of epithelial miRNAs in regulating ISC proliferation and facilitating intestinal regeneration. Mechanistically, the absence of canonical miRNAs, particularly the miR-200 family, leads to the accumulation of *Trp53* and *Cdkn1a* mRNAs, thereby triggering overactivation of the p53 pathway. Notably, similar findings are observed in colonic tissues derived from patients with ulcerative colitis (UC), where the downregulation of the miR-200 family and the hyperactivation of the p53 pathway are evident. Encouragingly, the oral administration of LNPs-miR-200 promotes the regeneration of the intestine in a mouse model following damage.

## Results and Discussion

### *Dgcr8* Deletion Curtails Intestinal Epithelium Regeneration after Irradiation Injury

To investigate the role of epithelial miRNAs in the intestinal epithelium, we generated *Villin-creERT2;Dgcr8*^*loxP/loxP*^ mice to specifically deplete DGCR8, an essential factor in the biogenesis of canonical miRNAs. Notably, the DGCR8 protein is highly expressed in the crypt cells. Efficient ablation of *Dgcr8* in the intestines was confirmed by tamoxifen injection (Figure S1A–C). Small RNA sequencing of the intestinal epithelium from wild-type (WT) and *Dgcr8* knockout (KO) mice demonstrated a significant suppression of miRNA biogenesis upon *Dgcr8* deletion (Figure S1D). qRT-PCR analysis further confirmed the reduced levels of miRNAs in the *Dgcr8* KO intestinal epithelium (Figure S1E). *Dgcr8* KO mice showed decreased body weight at day 4 postinjection (dpi), which recovered by 12 dpi (Figure S1F). However, there were no discernible morphological differences between the WT and *Dgcr8* KO mice.

**Figure 1:**
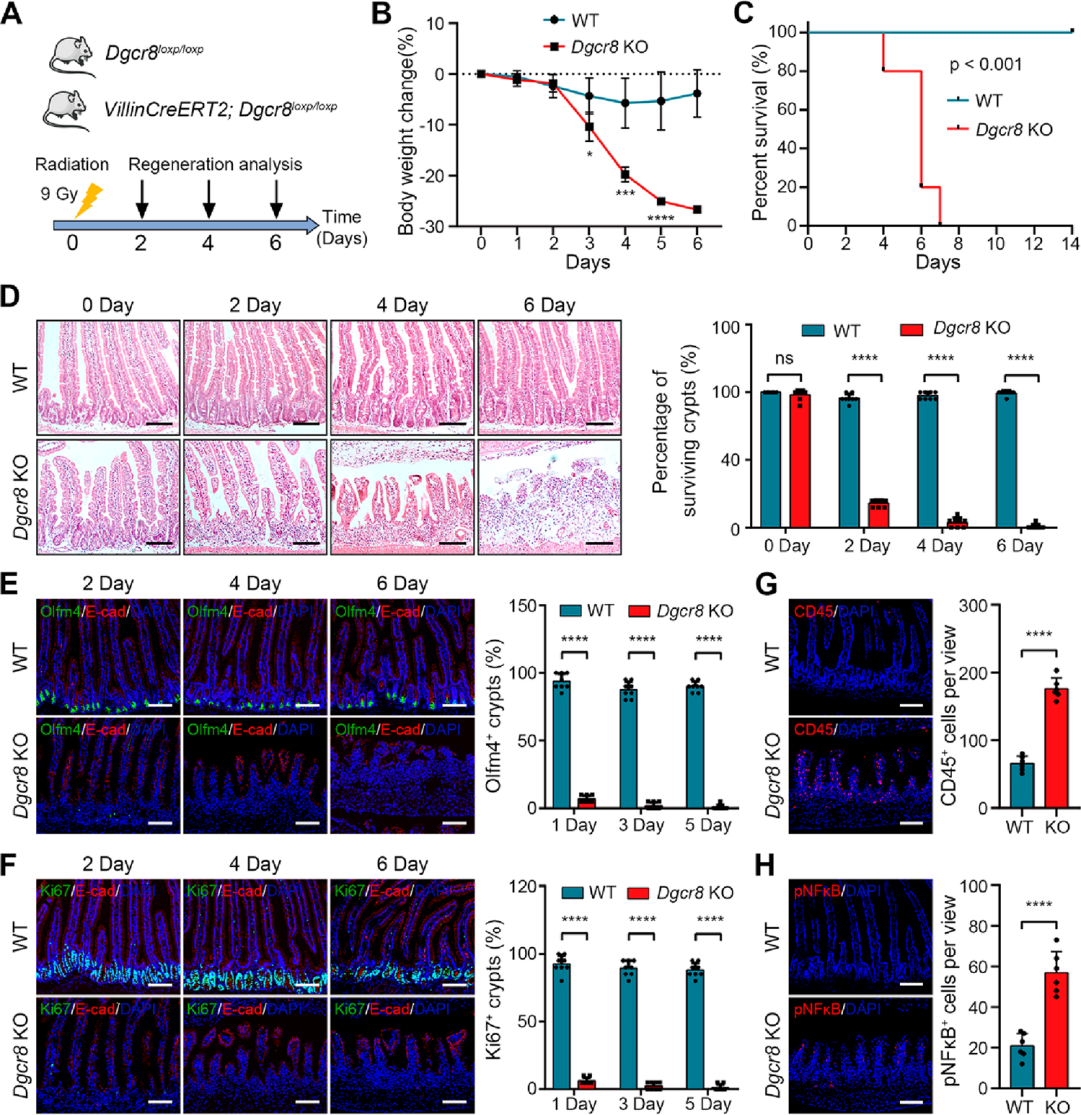
DGCR8 is indispensable for intestinal regeneration following irradiation. (A) Schematic diagram showing abdominal irradiation in WT and *Dgcr8* KO mice. (B,C) Body weight change and Kaplan–Meier survival curve of WT and *Dgcr8* KO mice after irradiation. *n* = 7 biological replicates for each genotype. (D) Histological images of small intestines (left) and percentage of surviving crypts (right) from WT and *Dgcr8* KO mice at indicated time points after irradiation. Images are representative of at least 3 mice at each time point for each genotype. (E,F) IF staining (left) and quantification (right) of Olfm4^+^ ISCs (E) and Ki67^+^ TA cells (F) in the intestinal epithelium from WT and *Dgcr8* KO mice. Epithelial cells were stained by E-cadherin (red). *n* ≥ 3 biological replicates for each genotype. (G,H) IF staining for CD45 and phosphorylated NF-κB (left) and quantification of CD45 and phosphorylated NF-κB positive cells (right) in intestinal epithelia from WT and *Dgcr8* KO mice 4 days after irradiation. *n* ≥ 3 biological replicates for each genotype. Statistics data represent mean with SD. *p*-values were generated by unpaired two-tailed Student’s *t*-test (B, D–H) and one-sided log-rank test (C). *p < 0.05; ***p < 0.001; ****p < 0.0001. Scale bars: 100 μm (D–H).

Next, we investigated the role of DGCR8 in intestinal regeneration following injury. Mice were exposed to a nonlethal dose of 9 Gy X-ray irradiation, known to induce damage in proliferating crypt epithelial cells,^19^ and crypt regeneration was assessed at days 2, 4, and 6 postirradiation (Figure 1A). *Dgcr8* KO mice in the intestinal epithelium exhibited significant weight loss and succumbed to death within a week, while control mice recovered quickly and survived (Figure 1B,C). In control mice, only a small number of crypts appeared shrunken at days 2–4 postirradiation and rapidly recovered from the injury (Figure 1D). In contrast, almost all crypts in *Dgcr8*-deficient intestinal epithelium shrank immediately after irradiation, and the villi shortened by day 4 postirradiation. IF analysis using the ISC marker Olfm4 revealed loss of ISCs in *Dgcr8*-deficient mice at day 2 postirradiation (Figure 1E). Cell proliferation marked by Ki67 was similarly impaired in KO mice following injury (Figure 1F). Additionally, *Dgcr8*-deficient mice exhibited increased levels of infiltration of CD45^+^ immune cells in the epithelium (Figure 1G), suggesting increased intestinal permeability. Moreover, overactivation of proinflammatory NF-κB signaling was observed in the epithelium of *Dgcr8* KO mice (Figure 1H). These findings underscore the crucial function of DGCR8 in crypt regeneration after injury.

### *Dgcr8* Deficiency Attenuates Intestinal Regeneration after 5-FU Treatment

**Figure 2:**
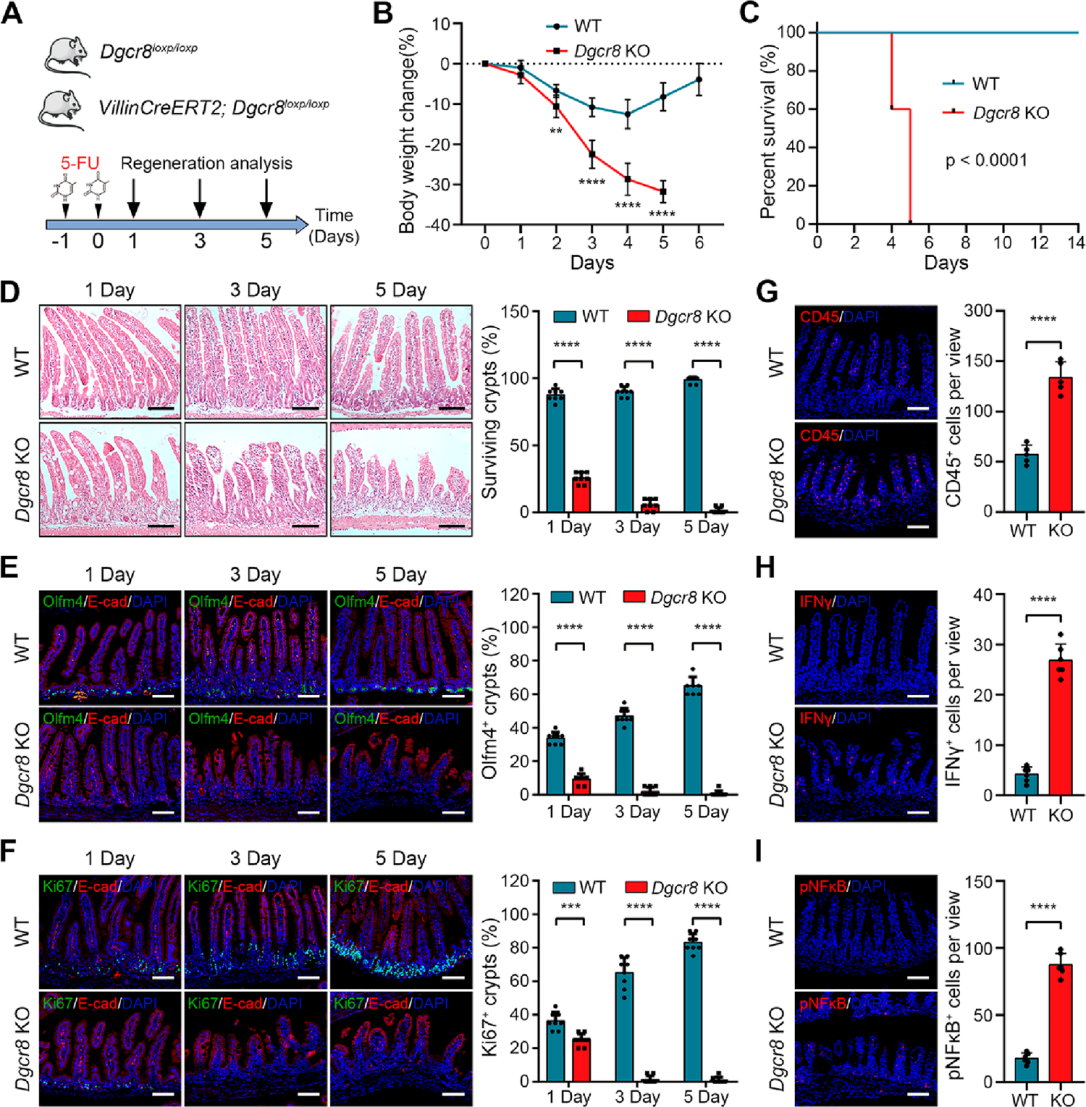
*Dgcr8*-deficient intestinal epithelium fails to regenerate after 5-FU induced injury. (A) Schematic diagram showing the 5-FU treatment in WT and *Dgcr8* KO mice. (B,C) Body weight change and Kaplan–Meier survival curve of WT and *Dgcr8* KO mice after 5-FU administration. *n* = 10 biological replicates for each genotype. (D) Histological images of small intestines (left) and percentage of surviving crypts (right) from WT and *Dgcr8* KO mice at indicated time points after 5-FU administration. Images are representative of at least 3 mice at each time point for each genotype. (E,F) IF staining (left) and quantification (right) of Olfm4^+^ ISCs (E) and Ki67^+^ TA cells (F) in the small intestinal epithelium from WT and *Dgcr8* KO mice. Epithelial cells were stained by E-cadherin (red). *n* ≥ 3 biological replicates for each genotype. (G–I) IF staining for CD45, IFNγ, and phosphorylated NF-κB (left) and quantification of CD45, IFNγ, and phosphorylated NF-κB positive cells (right) in intestinal epithelia from WT and *Dgcr8* KO mice 3 days after 5-FU administration. *n* ≥ 3 biological replicates for each genotype. Statistics data represent mean with SD. *p*-values were generated by unpaired two-tailed Student’s *t*-test (B, D–I) and one-sided log-rank test (C). **p < 0.01; ***p < 0.001; ****p < 0.0001. Scale bars: 100 μm (D–I).

To further validate the role of DGCR8 in intestinal regeneration, we employed another injury model using 5-Fluorouracil (5-FU) to induce the loss of proliferating epithelial cells^20^ (Figure 2A). Administration of 5-FU resulted in significant body weight loss in *Dgcr8*-deficient mice (Figure 2B), and all *Dgcr8*-deficient mice died within 5 days after injury (Figure 2C). Hematoxylin and Eosin (H&E) staining of the intestinal epithelium showed that *Dgcr8* ablation led to smaller crypts and villous atrophy (Figure 2D). IF staining revealed the loss of Olfm4^+^ ISCs shortly after injury in *Dgcr8*-deficient mice, whereas ISCs in WT mice initially decreased but quickly recovered (Figure 2E). Similarly, Ki67-positive proliferating cells in crypts of *Dgcr8*-deficient mice were lost shortly after injury, while TA cells in crypts of WT mice almost fully recovered within 5 days (Figure 2F). Furthermore, 5-FU treatment resulted in increased infiltration of CD45^+^ immune cells into the intestinal lumen in *Dgcr8*-deficient mice (Figure 2G), accompanied by elevated levels of the pro-inflammatory cytokine IFNγ and pNF-κB (Figure 2H,I). These phenotypes closely resemble the ones observed in the irradiated mice, providing further evidence that DGCR8 is essential for regenerating the intestinal epithelium.

### DGCR8 Is Required for Organoid Formation and ISC Maintenance

**Figure 3:**
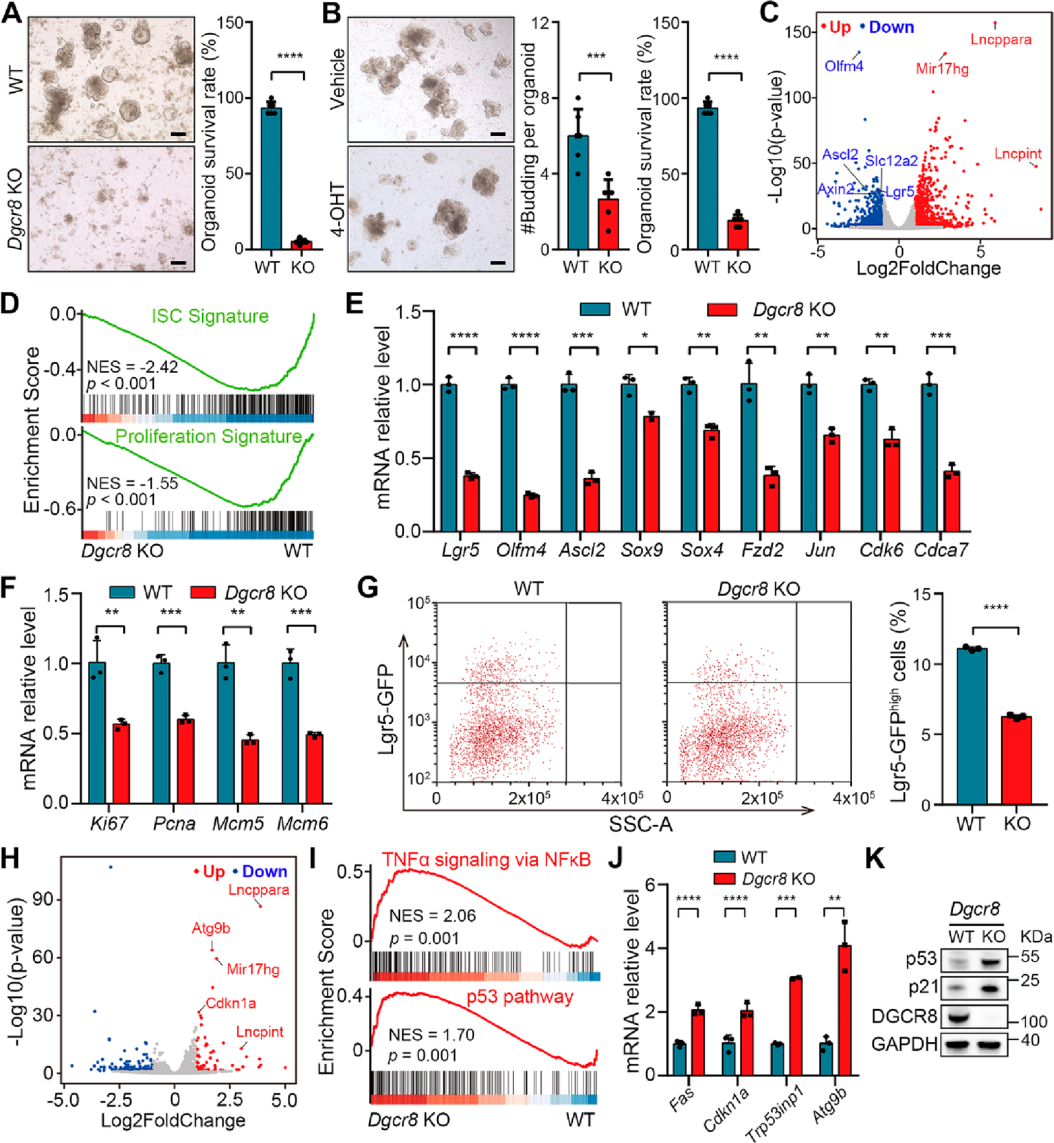
DGCR8 is required for intestinal organoid formation and ISC maintenance. (A) Representative images (left) and the number of surviving organoids per view (right) of intestinal organoids derived from WT and *Dgcr8* KO crypts. *n* ≥ 3 biological replicates for each genotype. (B) Representative images (left), quantification of budding numbers per organoid (middle), and survival rates (right) of *Villin-creERT2*;*Dgcr8*^*loxP/loxP*^ intestinal organoids cultured in medium with vehicle or 4-OHT. *n* = 6 biological replicates for each genotype. (C) Volcano plot showing the upregulated and downregulated genes in WT and *Dgcr8* KO intestinal organoids. (D) GSEA of ISC and proliferation signature gene sets enriched with decreased genes in *Dgcr8* KO intestinal organoids. NES, normalized enrichment score; *p*, nominal *p*-value. *p* < 0.05 was considered as statistically significant. (E,F) qRT-PCR analysis for ISCs and proliferation marker genes in WT and *Dgcr8* KO intestinal organoids. (G) FACS plots (left) and frequency quantification (right) of Lgr5-GFP^high^ ISCs in WT and *Dgcr8* KO intestinal organoids. *n* = 3 biological replicates for each genotype. (H) Volcano plot showing the upregulated and downregulated genes in WT and *Dgcr8* KO Lgr5-GFP^high^ ISCs. (I) GSEA of top 2 Hallmark gene sets enriched with increased genes in *Dgcr8* KO Lgr5-GFP^high^ ISCs. (J) qRT-PCR analysis for p53 target genes in WT and *Dgcr8* KO intestinal organoids. (K) Western blotting for p21, p53, DGCR8 and GAPDH in small intestines from WT and *Dgcr8* KO organoids. *n* = 3 biological replicates for each genotype. Statistics data represent mean with SD. *p*-values were generated by unpaired two-tailed Student’s *t*-test (A, B, E, F, G, J). *p < 0.05; **p < 0.01; ***p < 0.001; ****p < 0.0001. Scale bars: 200 μm (A, B).

To investigate the role of DGCR8 in ISCs, we generated intestinal organoids derived from *Dgcr8*-deficient and control mice.^21^ The crypts from the *Dgcr8*-deficient epithelium showed poor survival (Figure 3A). Similarly, the treatment of organoids derived from *Villin-creERT2;Lgr5-EGFP-IRES-creERT2;Dgcr8*^*loxP/loxP*^ mice with 4-OH-tamoxifen (4-OHT) resulted in decreased budding and increased cell death (Figure 3B).

Next, we performed bulk RNA sequencing on WT and *Dgcr8* KO organoids to uncover the regulatory mechanism of DGCR8. The expression of ISC marker genes such as *Lgr5*, *Olfm4*, *Ascl2*, and *Slc12a2*, was significantly downregulated upon *Dgcr8* inducible deletion. Conversely, several pri-miRNAs, including *Mir17hg*, *Lncppara*, and *Lncpint*, were upregulated in *Dgcr8*-deficient organoids (Figure 3C). Gene set enrichment analysis (GSEA) demonstrated that the *Dgcr8* deficiency reduced the expression of ISC and proliferation signature genes (Figure 3D), consistent with the downregulation of ISC and proliferation markers (Figure 3E,F). Flow cytometry analysis confirmed the reduction in the number of Lgr5-GFP^high^ ISCs in intestinal organoids due to *Dgcr8* deficiency (Figure 3G).

To gain further insights into how DGCR8 regulates ISCs, we sorted Lgr5-GFP^high^ ISCs from both WT and *Dgcr8* KO organoids and performed bulk RNA sequencing analysis. Similarly, pri-miRNAs such as *Mir17hg*, *Lncppara*, and *Lncpint* were upregulated in *Dgcr8*-deficient ISCs (Figure 3H), indicating the impaired processing of pri-miRNAs and subsequent miRNA biogenesis due to *Dgcr8* deletion. Interestingly, *Cdkn1a*, a gene associated with proliferation inhibition, and *Atg9b*, a gene associated with autophagy, were also upregulated in *Dgcr8*-deficient ISCs. GSEA analysis revealed the TNFα-NFκB and p53 pathways as the top-ranking pathways among the upregulated genes (Figure 3I). The increased activity of the p53 pathway in *Dgcr8*-deficient organoids could explain the reduction in the number of Lgr5^+^ ISCs and decreased cell proliferation (Figure S2A). Consistently, p53 target genes, including *Fas*, *Cdkn1a*, *Tp53inp1*, and *Atg9b*, were significantly upregulated in *Dgcr8* KO organoids (Figure 3J). Furthermore, the protein levels of p53 and p21 were increased in *Dgcr8*-deficient organoids (Figures 3K and S2B). These findings indicate that *Dgcr8* deficiency in organoids leads to a decrease in the number of ISCs, reduced cell proliferation, and activation of the p53 pathway.

### The p53 Pathway Is Overactivated in *Dgcr8*-Deficient Intestine

**Figure 4:**
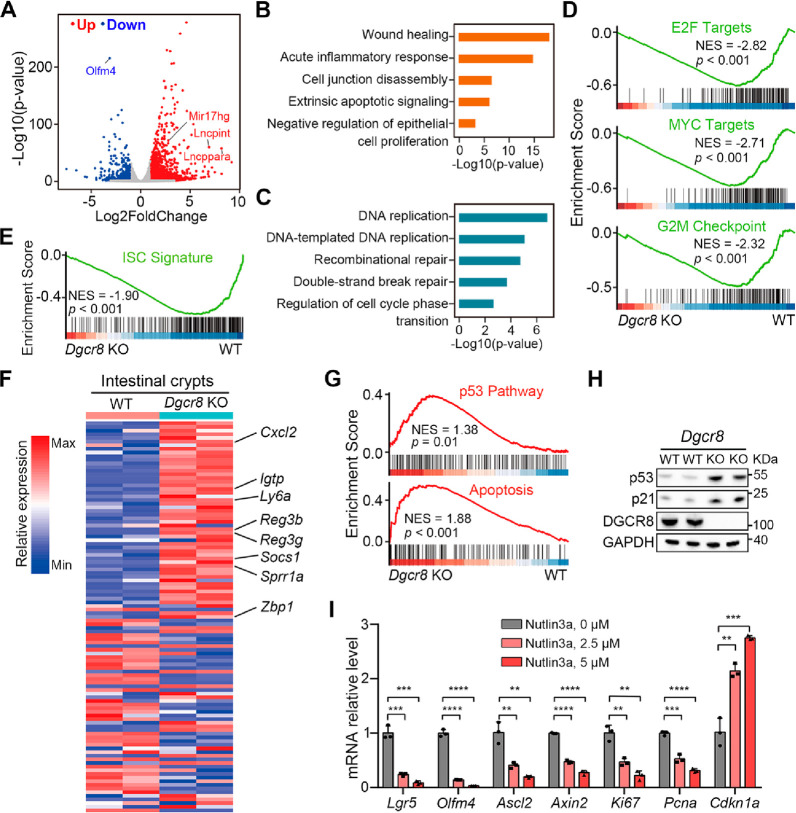
*Dgcr8* deficiency induces hyperactivation of the p53 pathway. (A) Volcano plot showing the upregulated and downregulated genes in crypts form WT and *Dgcr8* KO mice 1 day after 5-FU administration. (B,C) GO enrichment analysis of upregulated genes (B) and downregulated genes (C) in crypts from WT and *Dgcr8* KO mice 1 day after 5-FU administration, respectively. (D,E) GSEA of top Hallmark gene sets (D) and ISC signature gene set (E) enriched with decreased genes in crypts from *Dgcr8* KO mice 1 day after 5-FU administration. (F) Heatmap displaying the expression of an injury-associated regenerative signature genes in crypts from WT and *Dgcr8* KO mice 1 day after 5-FU administration. Representative genes are shown on the right. (G) GSEA of p53 pathway and apoptosis gene sets enriched with increased genes in crypts from *Dgcr8* KO mice 1 day after 5-FU administration. (H) Western blotting for p21, p53, and DGCR8 in small intestines from WT and *Dgcr8* KO mice 1 day after 5-FU administration. GAPDH was used as a loading control. (I) qRT-PCR analysis for ISC and proliferation marker genes in intestinal organoids incubated with different dose of MDM2 inhibitor nutlin3a. *n* = 3 biological replicates. Statistics data represent mean with SD. *p*-values were generated by unpaired two-tailed Student’s *t*-test (H,I). ns, not significant, *p* > 0.05; *p < 0.05; **p < 0.01; ***p < 0.001; ****p < 0.0001.

To confirm if p53 is also activated *in vivo*, we isolated crypts from both WT and *Dgcr8*-deficient mice 24 h after 5-FU administration and performed bulk RNA sequencing. The results showed that the pri-miRNAs *Mir17hg*, *Lncppara*, and *Lncpint* were upregulated in *Dgcr8*-deficient crypts, while the stem cell marker *Olfm4* was downregulated (Figure 4A). Gene Ontology (GO) enrichment analysis of differentially expressed genes (DEGs) indicated that the upregulated genes were significantly enriched in pathways related to the acute inflammatory response, apoptosis, and negative regulation of epithelial cell proliferation (Figure 4B). In contrast, the downregulated genes were found to be involved in DNA replication, double-strand break repair, and cell cycle phase transition (Figure 4C). GSEA analysis using Hallmark gene sets revealed that the downregulated genes were enriched in E2F targets, MYC targets, and the G2M checkpoint pathway, which are associated with cell cycle progression and cell proliferation (Figure 4D). These findings are consistent with the observed decrease in the level of proliferation marker Ki67 in the crypts of *Dgcr8*-deficient epithelium (Figure 2F).

Furthermore, GSEA analysis of the upregulated genes revealed enrichment in gene sets related to epithelial-mesenchymal transition, allograft rejection, inflammatory response, interferon-gamma response, TNF signaling via NF-κB, and IL6/JAK/STAT3 signaling (Figure S3A). The ablation of *Dgcr8* intensified the infiltration of CD45^+^ immune cells, production of the pro-inflammatory cytokine IFNγ, and activity of proinflammatory signaling pathways, including NF-κB and STAT3 (Figures 2G,I and S3B). These results highlight the role of DGCR8 in intestinal regeneration and suggest that DGCR8 deficiency may lead to an impaired regeneration capacity and increased inflammation in the intestine after injury.

ISC and proliferation signatures were enriched in the downregulated genes during the early phase of acute injury (Figures 4E and S3C). Importantly, the absence of epithelial miRNAs was associated with enrichment of the injury-associated signature (Figure 4F), which resembled the phenotype of the *Dgcr8*-deficient epithelium upon damage *in vivo*. Meanwhile, the p53 pathway and apoptosis were activated in the crypts of *Dgcr8*-deficient mice in response to 5-FU (Figure 4G). Immunoblotting confirmed elevated protein levels of both p53 and p21 in the *Dgcr8*-deficient epithelium (Figures 4H and S3D). These observations prompted further investigation into the role of the p53 pathway in regulating the ISC proliferation and epithelial regeneration. Treatment of intestinal organoids with the MDM2 inhibitor Nutlin3a, which stabilizes p53 protein, increased the expression of its direct target *Cdkn1a* mRNA and attenuated the expression of genes related to ISC and cell proliferation, reduced budding and increased cell death in a dose-dependent manner (Figures 4I and S3E). These findings support the involvement of the p53 pathway in the regulation of ISC proliferation and epithelial regeneration.

### Attenuation of the p53 Pathway Promotes Intestinal Regeneration in *Dgcr8*-Deficient Epithelium

**Figure 5:**
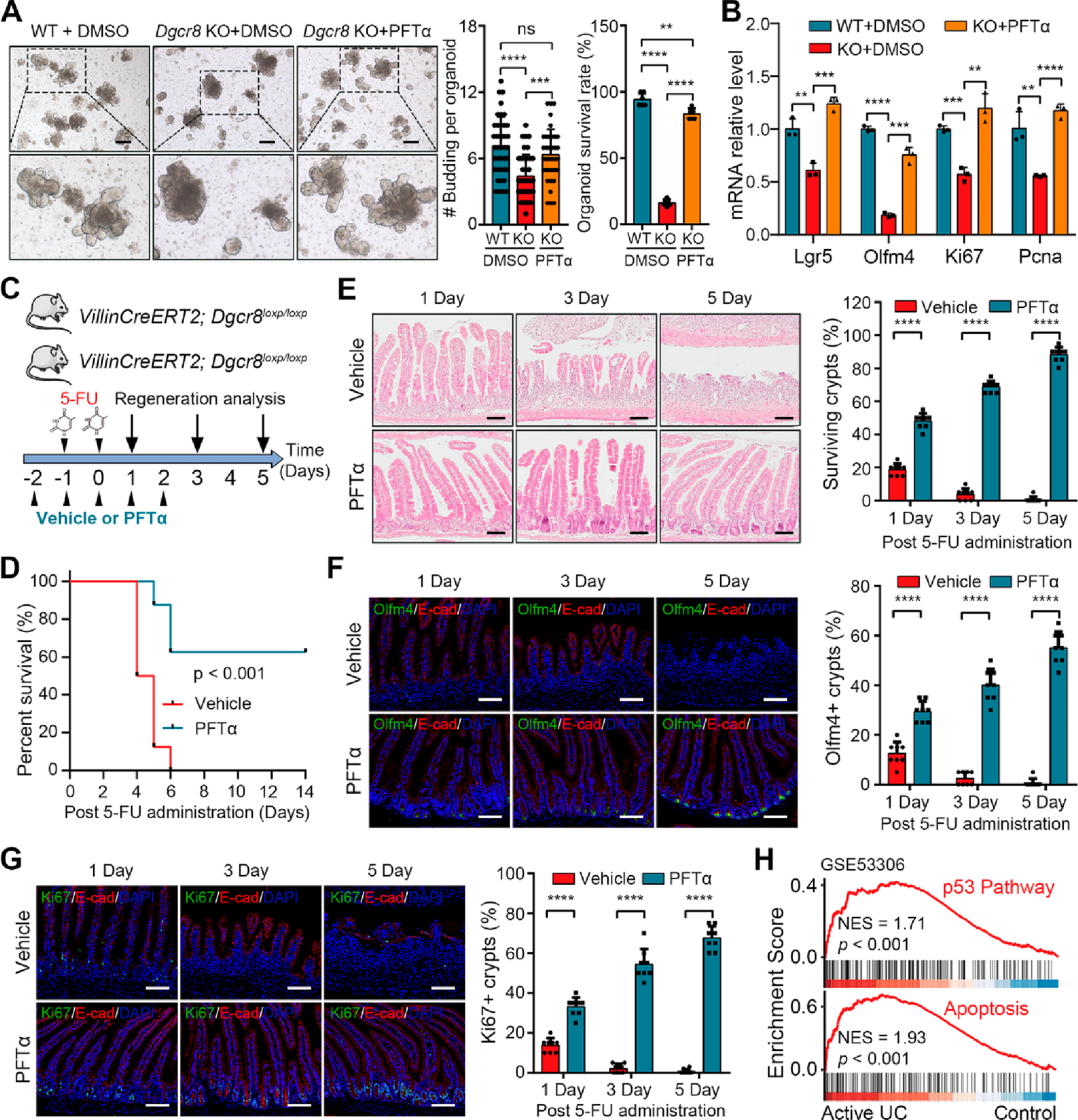
Inhibition of the p53 pathway promotes epithelial regeneration of *Dgcr8* deficient mice after 5-FU-induced injury. (A) Representative images (left), budding numbers (middle), and survival rates (right) of WT organoids with DMSO and *Dgcr8* KO organoids with DMSO or PFTα. The data represent mean ± SD derived from at least three wells per group. (B) qRT-PCR analysis for ISC and proliferation marker genes in WT organoids with DMSO and *Dgcr8* KO organoids with DMSO or PFTα. *n* = 3 biological replicates. (C) Schematic diagram showing the 5-FU treatment in *Dgcr8* KO mice along with PFTα or vehicle. (D) The Kaplan–Meier survival curve of *Dgcr8* KO mice after 5-FU administration with PFTα or vehicle. *n* = 8 biological replicates of each group. (E) Histological images of small intestines (left) and percentage of surviving crypts (right) from *Dgcr8* KO mice at indicated time points after 5-FU administration with PFTα or vehicle. (F,G) IF staining for Olfm4/Ki67 (left) and percentage of Olfm4^+^/ Ki67^+^ cells (right) in small intestinal crypts from *Dgcr8* KO mice at indicated time points after 5-FU administration with PFTα or vehicle. (H) GSEA enrichment of the p53 pathway and apoptosis gene sets in increased genes in colonic tissues from UC patients (GSE53306). Statistics data represent mean with SD. *p*-values were generated by unpaired two-tailed Student’s *t*-test (A, B, E–G) and one-sided log-rank test (D). ns, not significant; *p* > 0.05; **p < 0.01; ***p < 0.001; ****p < 0.0001. Scale bars: 200 μm (A), 100 μm (E–G).

Since the activity of the p53 pathway was negatively correlated with intestinal regenerative capacity, the effect of inhibiting the p53 pathway on intestinal regeneration was investigated. Treatment of *Dgcr8*-deficient organoids with the p53 protein inhibitor Pifithrin-α hydrobromide (PFTα) rescued the downregulation of genes associated with ISCs and proliferation, increased budding number, and improved organoid survival rate (Figure 5A,B). Furthermore, treatment with PFTα rescued 60% of the 5-FU treated *Dgcr8* KO mice from death (Figure 5C,D). Histological examination of the tissues showed that PFTα prevented the loss of crypts and effectively promoted intestinal regeneration following damage (Figure 5E), which was consistent with the rapid restoration of ISCs as shown by Olfm4 (Figure 5F). Additionally, PFTα was observed to restore TA cells in the *Dgcr8*-deficient epithelium (Figure 5G). These findings indicate that impaired intestinal regeneration associated with *Dgcr8* deficiency can be effectively rescued by inhibiting the p53 pathway.

To investigate the activation of the p53 pathway in damaged human intestinal epithelium, we analyzed publicly available data sets of colonic tissues from healthy individuals and patients with active ulcerative colitis (UC).^22,23^ The analysis revealed that the p53 pathway and apoptosis were activated in the colonic tissues of UC patients (Figures 5H and S4A). Additionally, the mRNA level of the antiproliferative and pro-apoptotic gene *TP53INP1* was positively correlated with the expression of pro-inflammatory cytokines interleukin (*IL*)*-1β* and *IL-6* in active UC tissues (Figure S4B). This provides evidence that hyperactivation of the p53 pathway is associated with the pathogenesis of colitis.

### Loss of MiR-200 Family Members Accounts for p53 Activation in *Dgcr8*-Deficient Cells

**Figure 6:**
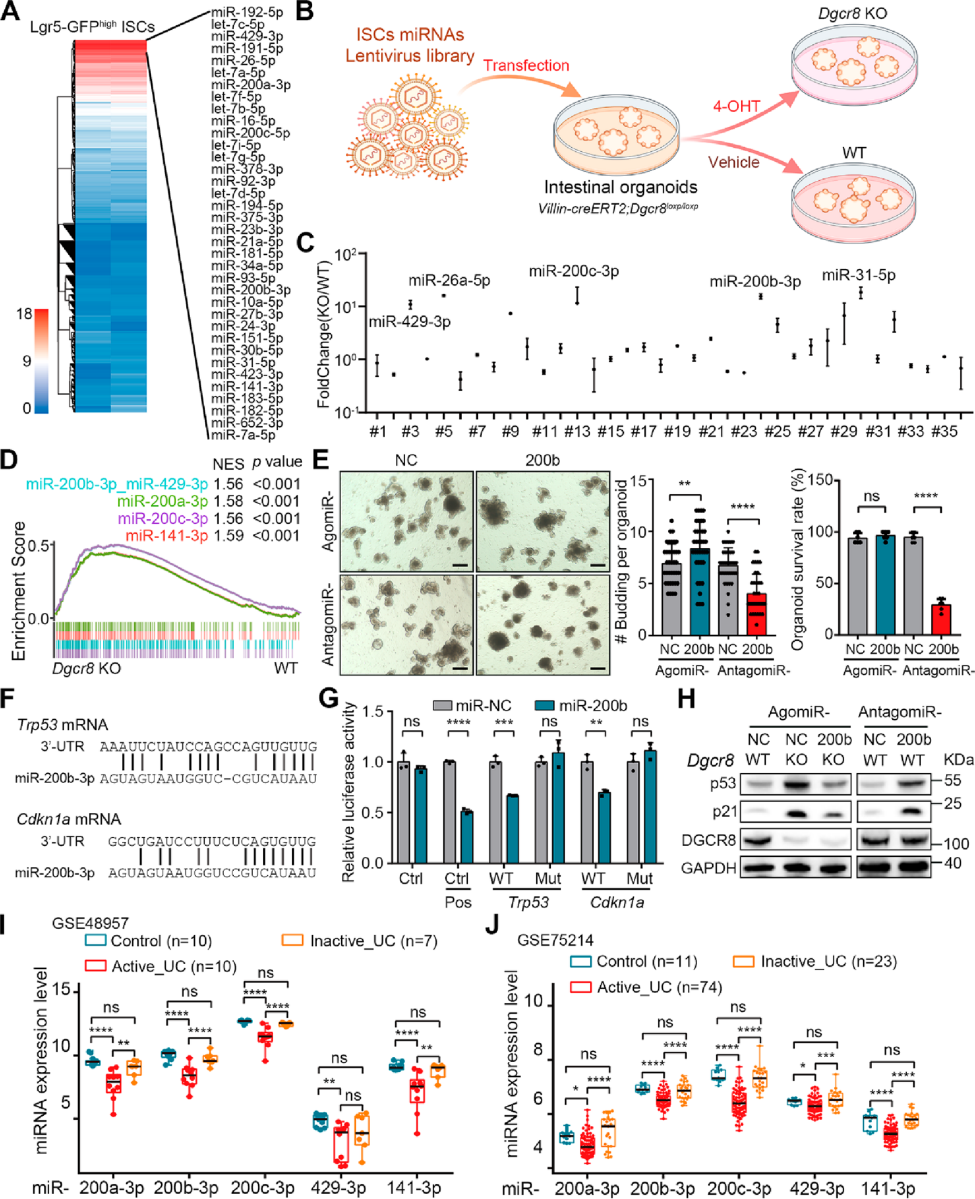
Loss of the miR-200 family contributes to hyperactivation of the p53 pathway in the intestinal epithelium. (A) Heatmap showing the relative expression level of miRNAs in GFP^high^ ISCs. (B) An illustration of a pooled miRNA function screen in organoids using a DGCR8-independent stable miRNA expression strategy. (C) qRT-PCR analysis for ISCs predominant miRNAs in *Villin-creERT2;Dgcr8*^*loxP/loxP*^ intestinal organoids transfected with ISCs predominant miRNAs overexpressing lentivirus relative to WT organoids transfected with ISCs predominant miRNAs overexpressing lentivirus. (D) GSEA enrichment of miR-200 family targeting gene set in increased genes in *Dgcr8* KO organoids. (E) Representative images (left), budding numbers (middle) and survival rates (right) of WT organoids transfected with agomiR-NC, agomiR-200b-3p, antagomiR-NC, or antagomiR-200b-3p. The data represent mean ± SD derived from at least three wells per group. (F) Predicted miR-200b-3p binding sites on the 3′-UTRs of *Trp53* and *Cdkn1a* mRNAs. (G) Relative luciferase activity of pmiR-reporter plasmids with or without WT and mutant miR-200b-3p binding sequence in cells transfected with agomiR-NC/200b-3p. (H) Western blotting for p21, p53 in WT organoids transfected with agomiR-NC/200b-3p or antagomiR-NC/200b-3p. GAPDH was used as a loading control. (I,J) Relative levels of miR-200 family members in colonic tissues from healthy controls and patients with inactive UC or active UC (GSE48957, GSE75214). *p*-values were generated by unpaired two-tailed Student’s *t*-test (E) and eBayes function in limma package (I,J). ns, not significant, *p* > 0.05; *p < 0.05; **p < 0.01; ***p < 0.001; ****p < 0.0001. Scale bars: 200 μm (E).

To identify the miRNAs responsible for the loss of ISCs and the activation of the p53 pathway in the *Dgcr8*-deficient epithelium and organoids, Lgr5-GFP^high^ ISCs were sorted from intestinal organoids and subjected to small RNA sequencing. Among the mapped miRNAs, only a few dozen were found to be highly expressed in ISCs (Figure 6A). When a cost-effective DGCR8-independent stable miRNA expression (DISME) strategy^24^ was employed to reintroduce the predominant miRNAs of ISCs back into *Villin-creERT2;Dgcr8*^*loxP/loxP*^ intestinal organoids (Figure 6B), the growth and survival of *Dgcr8*-deficient organoids were rescued. Repeated passaging of the organoids enriched essential miRNAs for growth and survival in the absence of *Dgcr8*. A pooled screen of miRNA function in organoids identified several miRNAs, including miR-31-5p, miR-200b-3p, miR-200c-3p, miR-26a-5p, and miR-429-3p, that were significantly enriched in the surviving *Dgcr8*-deficient organoids (Figure 6C). Among these miRNAs, miR-31 is known to drive ISC proliferation and promote epithelial regeneration following injury by regulating the activities of the Wnt and Hippo pathways.^14,15^ MiR-26a has been reported to repress NF-κB signaling and attenuate colitis.^25^ Members of the miR-200 family, including miR-200a-3p and miR-141–3p, were also enriched. The miR-200 family is a key regulator of epithelial-to-mesenchymal transition (EMT) by suppressing the expression of E-cadherin repressors ZEB1 and ZEB2,^26^ which is in agreement with the observation that the crypts showed activation of the EMT program in *Dgcr8*-deficient mice after 5-FU administration (Figure S3A). GSEA analysis further indicated that the potential targeting genes of the miR-200 family were significantly enriched in *Dgcr8*-deficient organoids (Figure 6D). Additionally, the transfection of a chemically modified miR-200b-3p agonist (agomiR-200b) promoted the budding process in organoids, while the transfection of a miR-200b-3p antagonist (antagomiR-200b) inhibited the budding process and reduced organoid survival (Figure 6E).

Interestingly, potential binding sites of miR-200b-3p were found on the 3′-UTRs of *Trp53* and *Cdkn1a* mRNAs (Figure 6F). The dual luciferase assay confirmed that miR-200b-3p could bind to these potential sites and suppress translation of the luciferase protein (Figure 6G). Transfection of agomiR-200b reduced p53 and p21 protein levels in *Dgcr8*-deficient organoids, while transfection of antagomiR-200b increased p53 and p21 protein levels (Figures 6H and S5A, B). Downregulation of five miR-200 family members was observed in colonic tissues from UC patients in multiple data sets (Figures 6I,J and S5C). Furthermore, a significant negative correlation has been observed between the expression of miR-200 family members and the pro-inflammatory cytokines *IL-1β* and *IL-6* in colonic tissues from UC patients (Figure S5D). Taken together, these findings indicate that deregulation of the miR-200 family members contributes to the hyperactivation of the p53 pathway, impaired epithelial regeneration, and increased inflammation.

### Delivery of MiR-200 via Lipid Nanoparticles Facilitates Intestinal Regeneration in Mice upon Damage

**Figure 7:**
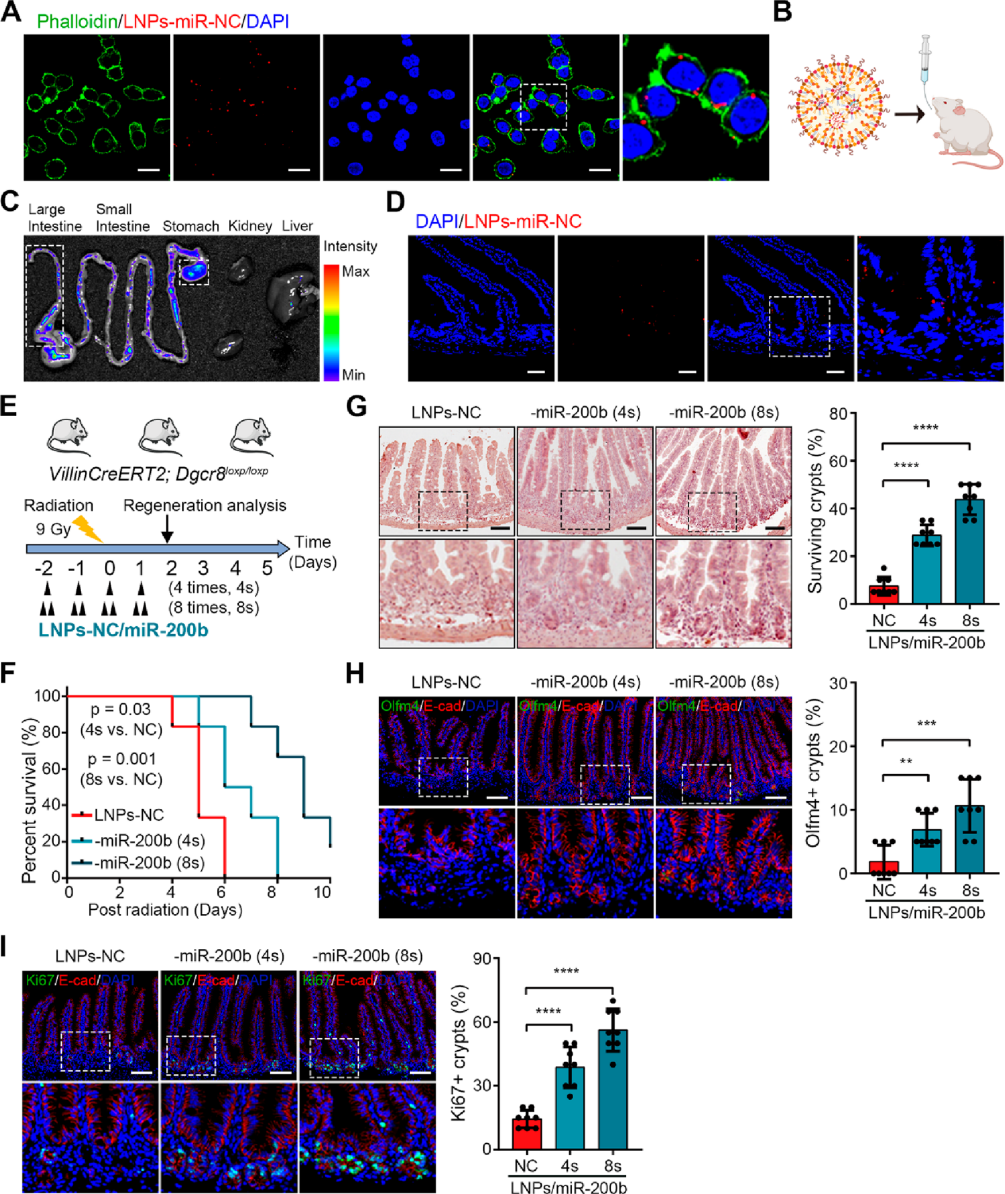
Oral delivery of miR-200 via lipid nanoparticles facilitates epithelial regeneration of *Dgcr8* deficient mice after irradiation-induced injury. (A) Representative images of Caco-2 cells transfected with LNPs-miR-NC-Cy3. (B) An illustration of oral delivery of LNPs-carrying miRNAs in mice. (C) Biodistribution of Cy3 in five organs (liver, kidney, stomach, small intestine, and large intestine) from mice received LNPs-miR-NC-Cy3 (3 mg/kg) by oral gavage. (D) Representative images of intestinal epithelium from mice treated with LNPs-miR-NC-Cy3 by gavage for 4 h. (E) Schematic diagram showing the irradiation treatment in *Dgcr8* KO mice along with oral gavage of 200 μL LNPs-NC/miR-200b (miR-200b, 1.5 mg/kg by body weight) once or twice a day for 4 consecutive days. (F) The Kaplan–Meier survival curve of *Dgcr8* KO mice after irradiation with gavage of LNPs-NC/miR-200b. *n* = 6 biological replicates of each group. (G) Histological images of small intestines (left) and percentage of surviving crypts (right) from *Dgcr8* KO mice after irradiation with gavage of LNPs-NC/miR-200b. (H,I) IF staining (left) and quantification (right) of Olfm4^+^ ISCs (H) and Ki67^+^ TA cells (I) in the intestinal epithelium from *Dgcr8* KO mice after irradiation with gavage of LNPs-NC/miR-200b. Statistics data represent mean with SD (G, H, I). *p*-values were generated by unpaired two-tailed Student’s *t*-test (G–I) and one-sided log-rank test (D). **p < 0.01; ***p < 0.001; ****p < 0.0001. Scale bars: 20 μm (A), 50 μm (D), 100 μm (G–I).

Given the capability of LNPs as carriers for nucleic acids and the demonstrated rescuing effect of miR-200 on the survival of *Dgcr8*-deficient organoids, we aimed to determine whether the delivery of miR-200 via lipid nanoparticles could promote intestinal regeneration in mice following damage. Initially, we generated LNPs-miR-200b by incorporating miRNA mimics into a lipid mixture using a microfluidic mixer. The polydispersity index (PDI) of all LNPs was less than 0.2, which indicated that they had a monodisperse state. The resulting LNPs-miRNAs exhibited a diameter of approximately 150–170 nm, with the zeta potential ranging from −5 to 0 mV and the encapsulation efficiency exceeding 75% (Figure S6A). Through fluorescence imaging of the human colon cancer cell line (Caco-2) transfected with LNPs-miR-NC-Cy3, miRNAs could be effectively delivered to cells by LNPs (Figure 7A). Next, mice were gavaged with Cy3 labeled LNPs-miR-NC and euthanized afterward to collect intestinal epithelial samples (Figure 7B). We observed successful delivery of miRNAs to intestinal epithelial cells using LNPs, and Cy3 labeled LNPs-miR-NC was mostly detected in the gastrointestinal tract (Figure 7C,D). MiR-200 levels were significantly increased in Caco-2 cells after incubation with LNPs-miR-200 and in intestinal epithelial cells in mice derived with oral gavage of LNPs-miR-200 (Figure S6B,C).

We then investigated whether delivering miR-200 via LNPs could alleviate the phenotypic changes within the intestinal epithelium of *Dgcr8*-deficient mice following damage induced by irradiation in a dose-dependent manner.^27^ LNPs-miR-200 reduced the extent of injury in the epithelium and rescued *Dgcr8* KO mice from death, evidenced by increased overall survival and a decrease in the number of damaged crypts in a dose-dependent manner (Figure 7E–G). The effective restoration of ISCs was observed with the use of LNPs-miR-200, as indicated by the expression of Olfm4 (Figure 7H). Similarly, TA cells within *Dgcr8*-deficient crypts were also restored by LNPs-miR-200 (Figure 7I). The protective effect of LNPs-miR-200 was also validated in *Dgcr8* KO mice with 5-FU administration (Figure S6D–H). These findings strongly suggest that delivering miR-200 via lipid nanoparticles has the potential to facilitate intestinal regeneration in irradiated or 5-FU-treated mice.

**Figure 8:**
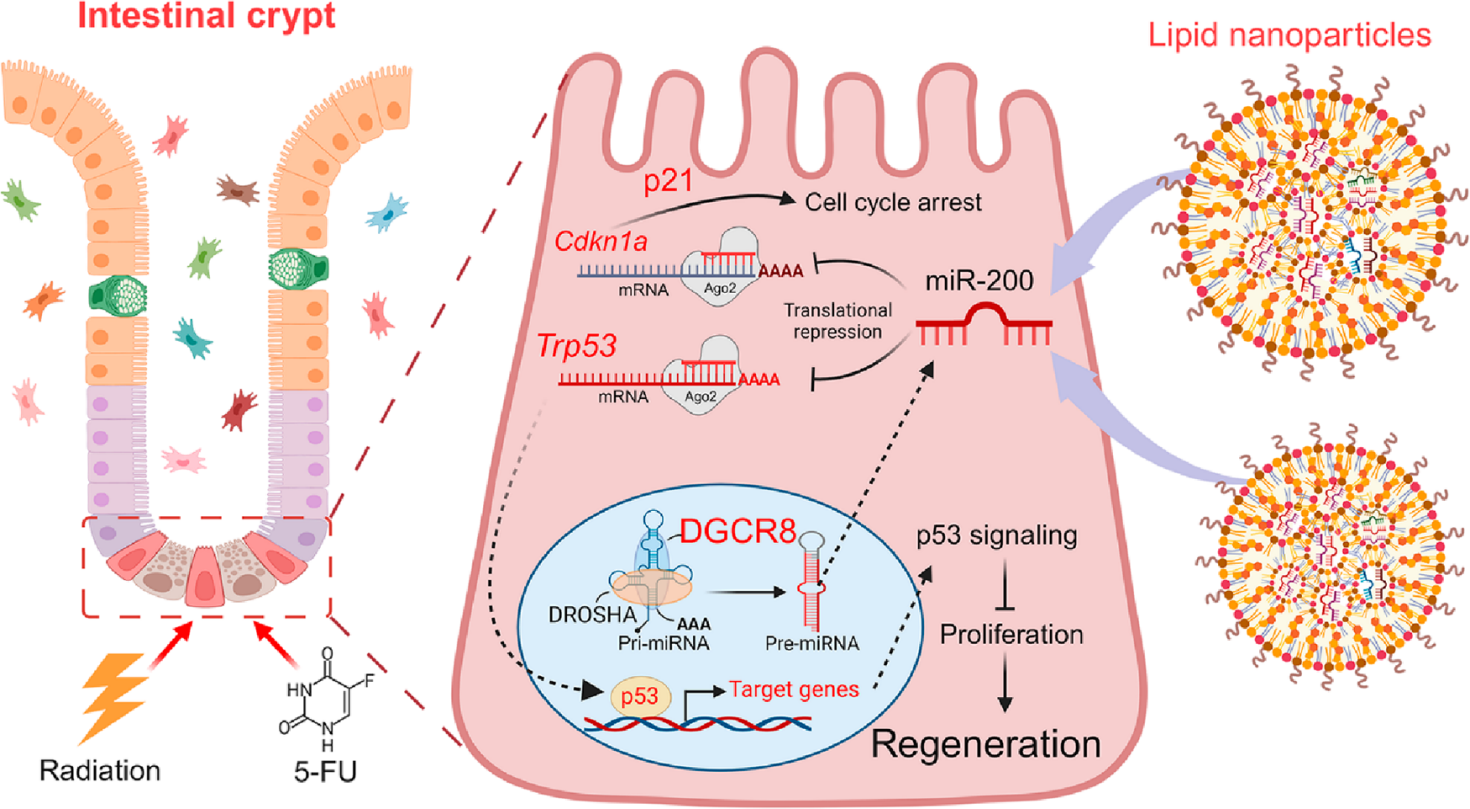
Schematic illustration demonstrates the role of lipid nanoparticles carrying miR-200 in promoting intestinal regeneration following acute injury in mice. In *Dgcr8*-deficient intestinal epithelial cells, the depletion of the miR-200 family, caused by the absence of *Dgcr8*, disrupts the precise regulation of Tr*p53* and *Cdkn1a* mRNA translation. Consequently, an elevated p53 pathway hampers the proliferation of ISCs and impedes epithelial regeneration. However, intestinal regeneration in mice is rescued by the delivery of miR-200 through lipid nanoparticles.

Our study highlights the crucial role of epithelial miRNAs in regenerating damaged intestinal epithelium. *Dgcr8* deficiency hyperactivates the p53 pathway, reducing ISC numbers and restricting cell proliferation, and accordingly attenuation of the p53 pathway rescues ISC loss and proliferation in *Dgcr8*-deficient organoids, partially restoring survival in mice with *Dgcr8*-deficient intestines after 5-FU treatment. The absence of *Dgcr8* impairs miRNA biogenesis in ISCs, and reintroducing predominant ISCs miRNAs identifies the miR-200 family as being essential for organoid survival. Notably, the miR-200 family directly binds to the 3′-UTRs of *Trp53* and *Cdkn1a* mRNAs, repressing their translation. Our findings reveal a regulatory mechanism, namely, the DGCR8/miR-200/p53 pathway, which plays a crucial role in intestinal regeneration. Importantly, we demonstrate that the transient restoration of miR-200 through oral delivery of LNPs carrying miR-200 promotes the process of intestinal regeneration in mice (Figure 8).

As a well-known tumor suppressor, p53 is a key regulator of homeostasis, regeneration, and the inflammatory response in the intestinal epithelium. Activation of Notch signaling and deletion of p53 in the intestinal epithelium leads to nuclear localization of Yap, enhances cell proliferation and regeneration, and promotes intestinal tumorigenesis with liver metastases.^28,29^ Treatment of p53 KO mice with DSS leads to colonic neoplasia.^30^ PUMA, a downstream target of p53, mediates apoptosis of ISCs, and its deficiency promotes crypt proliferation, regeneration, and survival after irradiation-induced damage.^31^ In response to irradiation-induced DNA damage, DGCR8 phosphorylation at serine 153 by JNKs and serine 677 by ATM facilitates transcription-coupled nucleotide excision repair in tumor cells, thereby enhancing cellular resistance to irradiation.^32,33^ The miR-200 family plays a critical role in promoting an epithelial state in cells.^34^ In mouse models of colitis, the levels of miR-200 family are correlated with the development of colitis.^35^ Decreased miR-141 expression is observed in colonic tissues of experimental colitis mouse models and IBD patients, and miR-141 has been shown to alleviate colitis in mouse models.^36^ Furthermore, miR-429 regulates mucin secretion and alleviates colitis in mouse models by targeting the *MARCKS* mRNA.^37^ These findings suggest that DGCR8 may regulate the p53 pathway through both canonical miRNA biogenesis and noncanonical DNA repair signaling pathways. However, the hyperactivation of the p53 signaling hinders epithelial cell proliferation, induces cell death, and curtails intestinal regeneration within damaged epithelium. The activity of p53 signaling could be finely regulated by epithelial-intrinsic and microenvironmental factors to restore epithelial homeostasis after acute injury. However, the role of the miR-200 family in intestinal regeneration in damaged epithelium is less appreciated.

LNPs-based drug delivery systems of small molecules, siRNA drugs and mRNA have been widely explored in preclinical studies for years.^38,39^ LNPs exhibit an excellent prospect of clinical translation in developing nanoparticles-based vaccines for COVID-19 and revolutionize the conventional strategy for vaccine development and implementation.^40^ In this study, we observed that the transient supply of miR-200 via LNPs promoted epithelial regeneration in *Dgcr8*-deficient mice following acute injury. Furthermore, oral delivery of LNPs-mRNA demonstrated protective effects in a mouse model of acute colitis induced by DSS.^18^ Additionally, LNPs-miRNA-193b exhibited significant delays in leukemia propagation in acute myeloid leukemia xenograft models, while LNPs containing miR-182 impaired tumor growth in triple-negative breast cancer models.^41,42^ Optimal LNPs should maximize the probability of reaching specific cell types within a target organ and minimize the toxicity and immunogenicity of cargoes.^43^ To enhance the stability, transfection efficacy, and safety of LNPs, various optimization strategies such as component selection, surface modification, suitable administration routes, and design of bioinspired compositions and structures have been explored.^44^ However, regarding the oral delivery of LNPs, more efforts are required to overcome the challenges presented by the complex digestive tract environment.^45^ Nonetheless, the restoration of the miR-200 family through a LNPs-based delivery system holds great promise for improving therapeutic efficacy in patients with enteritis or active UC. Advances in RNA engineering and delivery and LNP technology will facilitate the development of therapy for IBD patients.^46^

## Conclusions

Our study highlights the role of epithelial miRNAs in regulating ISC proliferation and promoting intestinal regeneration after acute injury through the DGCR8/miR-200/p53 axis. Additionally, we demonstrate that attenuation of the p53 pathway alleviates the repression of ISCs and the observed defects in epithelial regeneration. Importantly, LNPs carrying miR-200 facilitate intestinal regeneration. Our study emphasizes the crucial function of the DGCR8/miR-200/p53 axis in controlling intestinal regeneration and implies the use of miR-200-loaded LNPs as a promising therapeutic approach for patients with active UC, particularly those with reduced levels of the miR-200 family.

## Materials and Methods

### Mice

*Villin-creERT2* mice and *Lgr5-EGFP-IRES-creERT2* (Lgr5-GFP) mice were obtained from Jackson Laboratory. *Dgcr8*^*loxP/loxP*^ mice have been used.^11^ Male mice aged 2–4 months old, backcrossed into the C57BL/6 genetic lineage, were used for the study. To activate Cre recombinase, mice received intraperitoneal injections of 100 μL of Tamoxifen (20 mg/mL in corn oil, Sigma) for 5 consecutive days. Intestinal injury was induced in mice 14 days after Cre induction, through either 5-FU administration or irradiation. For 5-FU administration, mice were given intraperitoneal injections of 5-FU (120 mg/kg by body weight, MedChemExpress) for 2 consecutive days. For irradiation, mice were exposed to a single fraction of abdominal irradiation (9 Gy). To perform rescue assays in mouse models, mice were intraperitoneally injected with PFTα (2.2 mg/kg by body weight, MedChemExpress) or gavaged with LNPs carrying miRNA mimics (1.5 mg/kg by body weight, RiboBio) for 5 consecutive days. Mice were analyzed at specified time points following an intestinal injury. All animal experimental procedures were conducted in accordance with the appropriate guidelines and approved by the ethical committee for the use of laboratory animals at the Guangzhou Institute of Biomedicine and Health, Chinese Academy of Sciences.

### Intestinal Crypt Isolation and Organoid Culture

The murine small intestines were longitudinally incised and rinsed with cold PBS. The villi were carefully removed, and the intestines were cut into 5 mm pieces. These pieces were then incubated in cold PBS containing 2 mM EDTA for 30 min on ice. Afterward, the pieces were vigorously suspended in cold PBS, and the resulting supernatant was passed through a 70 μm cell strainer (Corning). The crypts were centrifuged (5 min at 300g), embedded in Matrigel (Corning), and seeded in 24-well plates. The crypts embedded in Matrigel were cultured in ENR medium consisting of DMEM/F12 (Gibco), EGF (50 ng/mL, Invitrogen), Noggin (100 ng/mL, R&D), R-spondin1 (500 ng/mL, R&D), and supplemented with Penicillin/Streptomycin, GlutaMAX, N2, B27, and *N*-acetylcysteine (Gibco). To induce Cre expression in transgenic organoids, 4-OHT (200 nM, Sigma) was added to the culture medium for 24 h after the initial passage of the organoids.

### Synthesis and Characterization of Lipid Nanoparticles (LNPs)

The ethanol phase contained a mixture of Dlin-MC3-DMA, cholesterol, DOPE, and DMG-PEG 2000 at 35:46.5:16:2.5. The aqueous phase was prepared by using citrate buffer with miRNA. LNPs@miRNA was formulated by mixing the aqueous phase with the ethanol phase. Resultant LNPs were dialyzed in PBS at 4 °C for 2 h. The miRNA encapsulation efficiency was tested by a Quant-iT RiboGreen RNA Quantitation Kit. LNP@miRNA was treated with TE buffer (measuring unencapsulated miRNA) or TE buffer with 1% Triton X-100 (measuring total miRNA). The Ribogreen reagent was added, and the fluorescence signal was measured. The standard curve was used to quantify the RNA content and calculate the encapsulation efficiency. Encapsulation efficiency (EE%) was calculated as follows: EE% = ((1 – unencapsulated miRNA)/total miRNA) × 100%. The diameter and zeta potential of the nanoparticles were analyzed using a Zetasizer Nano ZS instrument (Malvern Instruments, Malvern, U.K.).

### Lgr5-GFP^high^ Cell Isolation and Flow Cytometry

Following passaging, *Villin-creERT2;Lgr5-EGFP-IRES-creERT2;Dgcr8*^*loxP/loxP*^ intestinal organoids were treated with either 4-OHT or vehicle for 24 h and then cultured in ENR medium for an additional 3 days. The organoids were collected and incubated with TryPLE (Invitrogen) at 37 °C for 15 min to dissociate the cells. After dissociation, the cells were filtered through 40 μm cell strainers (Corning), and single Lgr5-GFP^high^ cells were sorted using a FACS Aria III Cell Sorter (BD Bioscience) based on the GFP signal. For flow cytometry analysis of ISCs, the disaggregated single cells were analyzed for GFP signal using a flow cytometer (CytoFLEX, Beckman).

### RNA Isolation and Quantitative Real-Time PCR

Total RNA was extracted from crypts, organoids, and Lgr5-GFP^high^ cells using the TRIzol Reagent (Life Technologies) according to the manufacturer’s instructions. Subsequently, cDNA was synthesized from 1 μg of total RNA using the HiScript III RT SuperMix (Vazyme). Quantitative real-time PCR (qRT-PCR) was performed on a QuantStudio 3 Real-Time PCR System (Applied Biosystems) using the ChamQ SYBR qPCR Master Mix (Vazyme) and specific primers. The expression levels were normalized to a housekeeping control gene, and the primer sequences used are provided in the Supplementary Tables.

### Bulk RNA Sequencing Data Analysis

Following total RNA extraction, library construction was carried out based on the Illumina HiSeq platform with two biological replicates. The bulk RNA sequencing was conducted by Novogene and Annoroad Gene Technology. The resulting clean reads were aligned to the mm10 mouse genome reference (GRCh38) using the STAR aligner with default parameters.^47^ The R package DESeq2^48^ was employed to identify DEGs using the gene counts data obtained from STAR. DEGs were defined as genes with an absolute Log2FoldChange >1 and a *p*-value <0.05. GO enrichment analysis of the DEGs was performed using the R package ClusterProfiler.^49^ Additionally, GSEA (Gene Set Enrichment Analysis) was conducted using the R package ClusterProfiler to assess the enrichment of gene sets related to ISC,^50^ proliferation,^51^ epithelial cell proliferation signature genes, and Hallmarks.

### Small RNA Sequencing Data Analysis

For small RNA sequencing data analysis, the miRNAs of WT and *Dgcr8* KO crypts were obtained from miRBase Release 22.1,^52^ and miRNA references were constructed using Bowtie2 v2.2.5.^53^ Subsequently, Mirdeep v2.0.1.2 was employed to align fastq files to the miRNAs of crypts and to quantify them.^54^ And, mall RNA sequencing data of Lgr5-GFP^high^ cells was analyzed on the Dr. Tom Multiomics Data Mining System (BGI). The clean data was mapped to the reference genome and small RNA database including miRbase with Bowtie2, and miRNA expression level was calculated by counting absolute numbers of molecules using molecular identifiers.

### Immunoblotting

Intestinal organoids, epithelial tissues, and crypts were collected and lysed in RIPA lysis buffer (Beyotime) supplemented with a protease and phosphatase inhibitor cocktail (Roche) to extract proteins. The protein lysates were then separated by SDS-PAGE, and the blots were incubated with primary antibodies overnight at 4 °C. Primary antibodies used included rabbit anti-DGCR8 (Abcam, ab191875, 1:1000), mouse anti-p53 (CST, 2524, 1:1000) and rabbit anti-p21 (Abcam, ab188224, 1:800). To detect GAPDH as a loading control, a rabbit anti-GAPDH (CST, 5174, 1:2000) antibody was used. Following this, the protein blots were incubated with corresponding secondary antibodies at room temperature for 2 h.

### Hematoxylin and Eosin (H&E), Immunofluorescence (IF), and Immunohistochemistry (IHC)

The mouse intestines were washed with PBS and then fixed in 4% paraformaldehyde (Sigma). After fixation, the intestines were embedded in paraffin, and 5-μm-thick sections were prepared for H&E staining and immunofluorescence (IF) staining. H&E staining was performed using an H&E Staining Kit from Sangon Biotech according to the manufacturer’s instructions. The stained sections were then imaged using an Axio Imager A2 imaging system (ZEISS). For IF staining, the sections were subjected to antigen retrieval using sodium citrate buffer. Permeabilization was achieved by treating the sections with a permeabilizing agent. To block nonspecific binding, the sections were incubated in a PBS solution containing 0.3% Triton X-100 and 5% BSA for 1 h at room temperature. Next, the sections were incubated overnight at 4 °C with primary antibodies: rabbit anti-Ki67 (Abcam, ab15580, 1:1000), rabbit anti-Olfm4 (CST, 39141, 1:500), rabbit anti-Lysozyme (Abcam, ab108508, 1:500), mouse anti-E-Cadherin (CST, 14472, 1:1000), rabbit anti-Muc2 (Abcam, ab272692, 1:1000), rabbit anti-Chromogranin A (Abcam, ab15160, 1:500), rabbit anti-CD45 (Abcam, ab10558, 1:1000), mouse anti-IFN-γ (Santa Cruz, sc-8423, 1:100), rabbit anti-Phospho-NF-κB p65 (CST, 3033, 1:400), or rabbit anti-Phospho-Stat3 (CST, 9145, 1:500). After incubation with the primary antibodies, the sections were incubated with Alexa Fluor 488-, Alexa 594-, and Alexa 647-conjugated secondary antibodies (Invitrogen, 1:1000) for 2 h at room temperature. Human colon cancer cells were fixed by 4% PFA, permeabilized, and incubated with Phalloidin (Beyotime, C2201S, 1:500) for 0.25 h. Nuclei were counterstained with DAPI (Sigma, D8417). For IHC staining, sections were quenched with 0.3% H_2_O_2_ after antigen retrieval. Sections were incubated with a secondary horseradish peroxidase conjugated antibody for 2 h and then with primary antibody anti-DGCR8 (Abcam, ab191875, 1:500) overnight at 4 °C. Finally, slides were dehydrated and mounted with neutral balsam. Imaging of the stained sections was performed using either an LSM 800 Confocal Laser Scanning Microscope (ZEISS) or an FV3000 Confocal Laser Scanning Microscope (Olympus).

### MiRNA Agomir and Antagomir

Chemically modified miRNA agonist and antagonist, referred to as miRNA agomir and antagomir, respectively, were obtained from RiboBio and incubated with organoids for 2 days at a concentration of 10 nM, following the manufacturer’s instructions.

### Statistical Analysis

To ensure the accuracy and reliability of the study results, all experiments were conducted independently at least three times. In vivo analyses were performed using a minimum of three animals per condition, as indicated in the figure legends. Bulk RNA sequencing was performed with two biological replicates. For organoid analysis, only organoids with a diameter of ≥100 μm were selected from each group and cultured in triplicate. Statistical analysis was conducted using GraphPad Prism version 7. Two-tailed unpaired Student’s *t* test was used to determine the statistical significance between groups, and *p*-values were generated accordingly. The specific *p*-values obtained from the statistical analysis can be found in the corresponding results or figure captions.

## Data Availability

The bulk-RNA sequencing data and the small RNA sequencing data have been deposited in the Gene Expression Omnibus (GEO) database (GSE230172). The ulcerative colitis data sets with accession codes GSE75214, GSE53306, GSE48957, and GSE66932 can be accessed on the GEO Web site (http://www.ncbi.nlm.nih.gov/geo).

## Supporting Information Available

Deletion of *Dgcr8* disrupts miRNA biogenesis in the intestinal epithelium; *Dgcr8* deficiency results in activation of p53 signaling and represses proliferation-associated genes in intestinal organoids; *Dgcr8* deficiency induces the hyperactivation of mucosal immunity in the intestinal crypts within damaged epithelium; Activation of the p53 pathway and apoptosis in the colonic tissues from patients with active UC; Downregulation of the miR-200 family in colonic tissues correlates with the pathogenesis of UC; Oral delivery of miR-200 via lipid nanoparticles promotes epithelial regeneration of Dgcr8-deficient mice after 5-FU-induced injury; Primers for qRT-PCR of mRNAs; Primers for reverse transcription of miRNAs; Primers for quantitative real-time PCR of miRNAs; Primers for miR-200b-3p binding sites in the dual luciferase reporter assay (PDF)
