# Supplementary material for: MicroRNA-200 Loaded Lipid Nanoparticles Promote Intestinal Epithelium Regeneration in Canonical MicroRNA-Deficient Mice
Source: ACS Nano. 2023 Nov 8;17(22):22901–15. doi: 10.1021/acsnano.3c08030 (PMC10690841; doi:10.1021/acsnano.3c08030)
Supplement: Supplementary file 1 — nn3c08030_si_001.pdf [file nn3c08030_si_001.pdf]

Supporting Information

MicroRNA-200 loaded lipid nanoparticles promote intestinal epithelium regeneration in canonical microRNAs-deficient mice

Xiyang Wei[1,2], Shicheng Yu[1,2], Tinghong Zhang[2], Liansheng Liu[1,2], Xu Wang[2], Xiaodan Wang[3], Yun-Shen Chan[2], Yangming Wang[4], Shu Meng[2], Ye-Guang Chen[2,3,5,*]

[1]Guangzhou Institutes of Biomedicine and Health, Chinese Academy of Sciences, Guangzhou 510530, China

[2]Guangzhou National Laboratory, Guangzhou 510005, China

[3]The State Key Laboratory of Membrane Biology, Tsinghua-Peking Center for Life Sciences, School of Life Sciences, Tsinghua University, Beijing 100084, China

[4]Institute of Molecular Medicine, College of Future Technology, Peking University, Beijing 100871, China

[5]School of Basic Medicine, Jiangxi Medical College, Nanchang University, Nanchang 330031, China

[*]Correspondence: ygchen@tsinghua.edu.cn (Ye-Guang Chen)

Supplementary Figures 1-6

Supplementary Tables 1-4

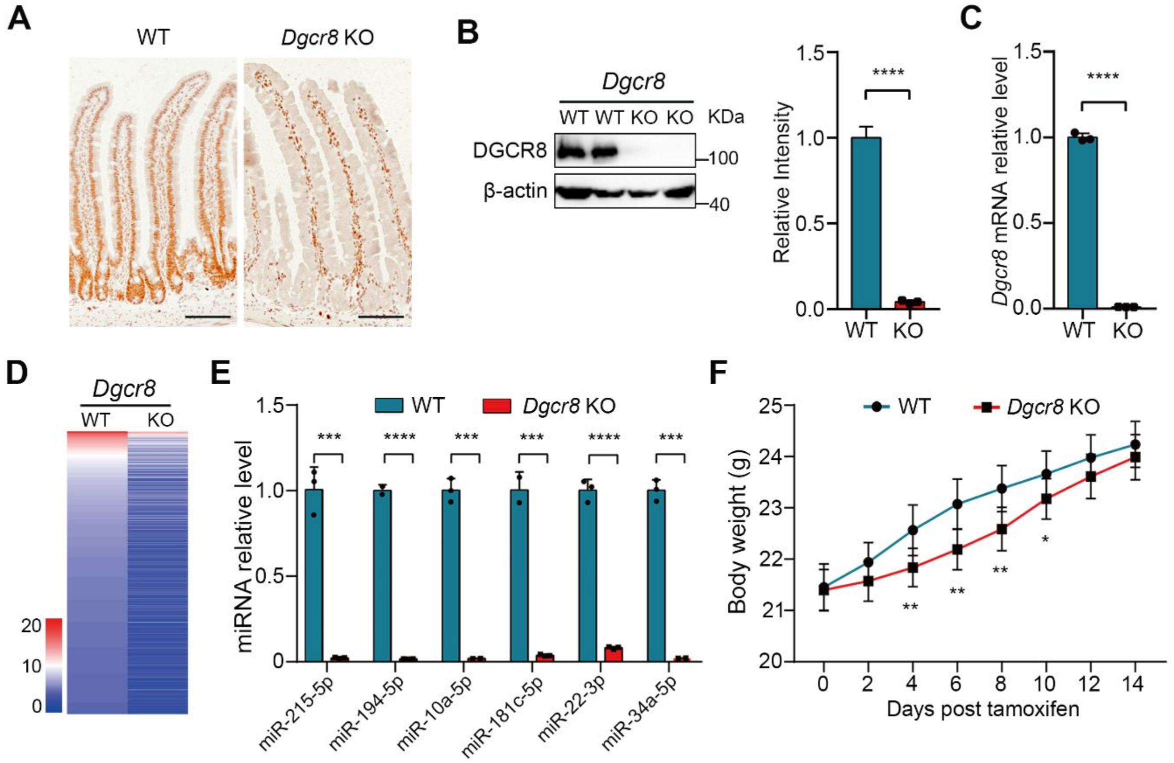

Figure S1. Deletion of *Dgcr8* disrupts miRNA biogenesis in the intestinal epithelium. (A) Representative images of DGCR8 IHC staining with antibody in small intestines from WT and *Dgcr8* KO mice. (B,C) DGCR8 protein (B) and *Dgcr8* mRNA (C) in the intestinal epithelium from WT and *Dgcr8* KO mice. (D) Heatmap showing the relative expression of miRNAs in the intestinal epithelium from WT and *Dgcr8* KO mice. (E) qRT-PCR analysis for relative levels of selected miRNAs in the intestinal epithelium from WT and *Dgcr8* KO mice. n = 3 biological replicates. (F) Body weight of WT and *Dgcr8* KO mice at designated time points after intraperitoneal injection of tamoxifen. n = 8 biological replicates for each genotype. Statistics data represent mean with standard deviation (SD). All p values were generated by unpaired two-tailed Student's t-test. *p<0.05; **p<0.01; ***p<0.001; ****p<0.0001. Scale bar: 100 μm.

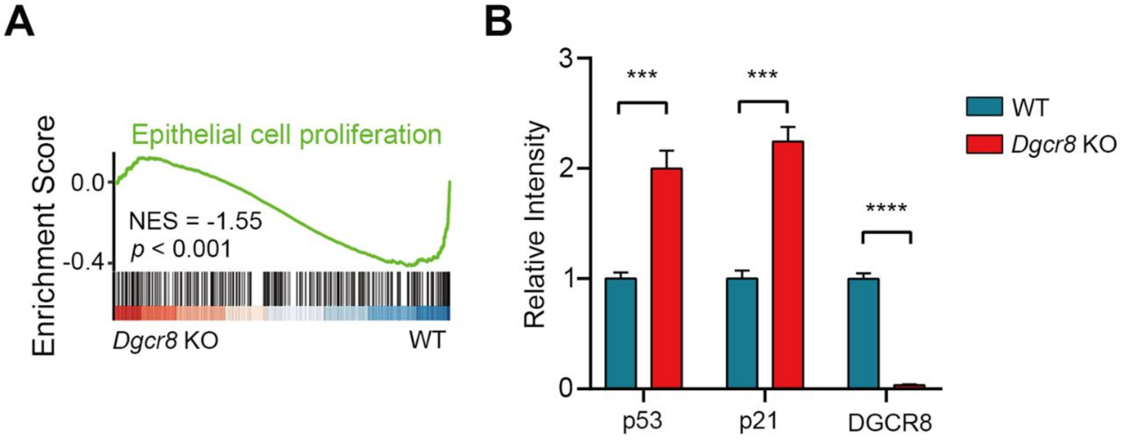

Figure S2. *Dgcr8* deficiency results in activation of p53 signaling and represses proliferation-associated genes in intestinal organoids. (A) GSEA of epithelial cell proliferation gene set enriched with decreased genes in *Dgcr8* KO Lgr5^{high} ISCs. (B) Quantification of western blot for p21, p53, DGCR8 in WT and *Dgcr8* KO intestinal organoids (Figure 3K). Statistics data represent mean with SD. *p* values were generated by unpaired two-tailed Student's *t*-test (B). ***p<0.001; ****p<0.0001.

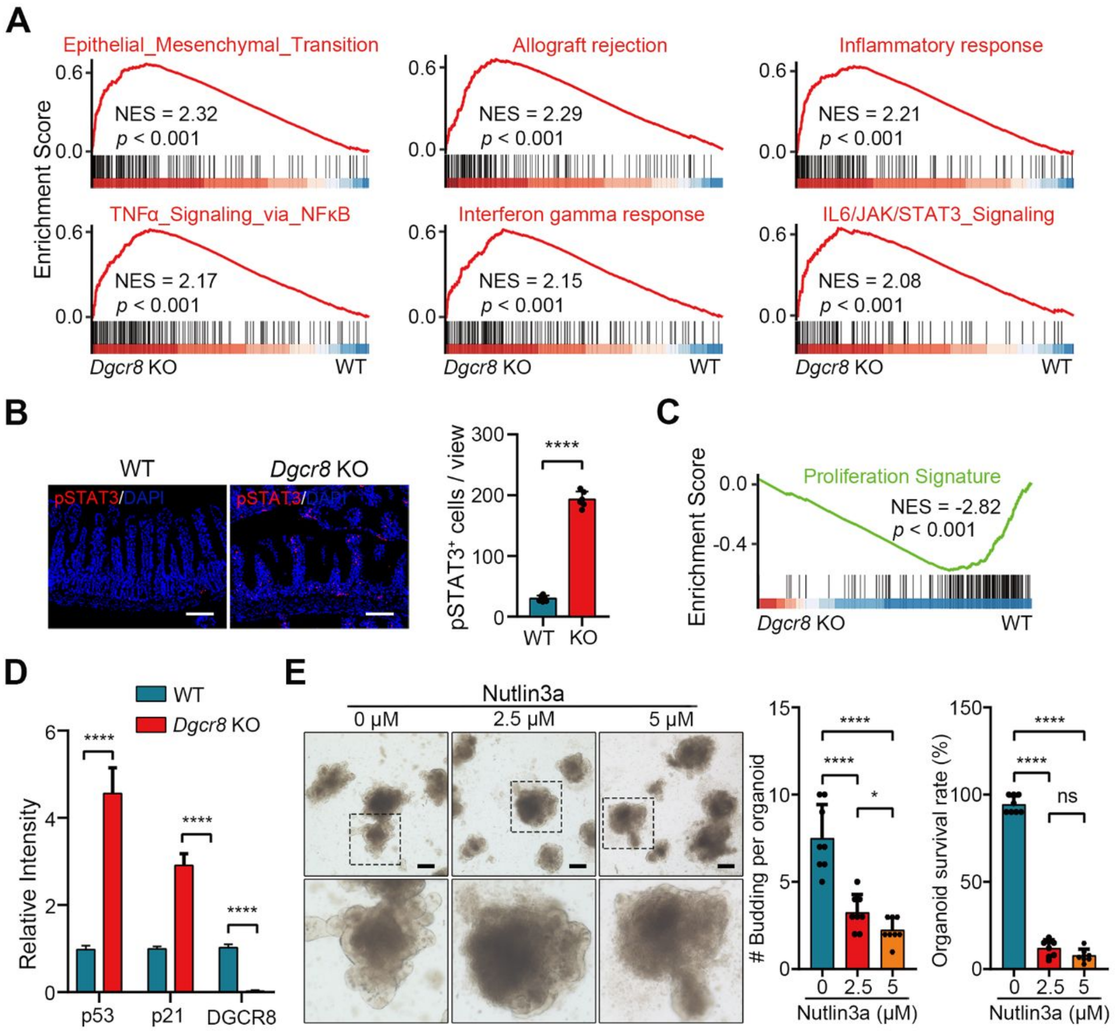

Figure S3. *Dgcr8* deficiency induces the hyperactivation of mucosal immunity in the intestinal crypts within damaged epithelium. (A) GSEA of top hallmark gene sets enriched with increased genes in small intestinal crypts from *Dgcr8* KO mice 1 day after 5-FU treatment. (B) IF staining for phosphorylated STAT3 (left) and percentage of phosphorylated STAT3 positive cells (right) in intestinal epitheliums from WT and *Dgcr8* KO mice 3 days after 5-FU treatment. (C) GSEA of proliferation signature gene set enriched with decreased genes in crypts from *Dgcr8* KO mice 1 day after 5-FU administration. (D) Quantification of western blot for p21, p53, DGCR8 in WT and *Dgcr8* KO intestines (Figure 4H). (E) Representative images (left), budding numbers (middle) and survival rates (right) of WT organoids treated with nutlin3a. Statistics data represent mean with SD. p value was generated by unpaired two-tailed Student's t-test (B,D). ****p<0.0001. Scale bars: 100 μm (B), 200 μm (E).

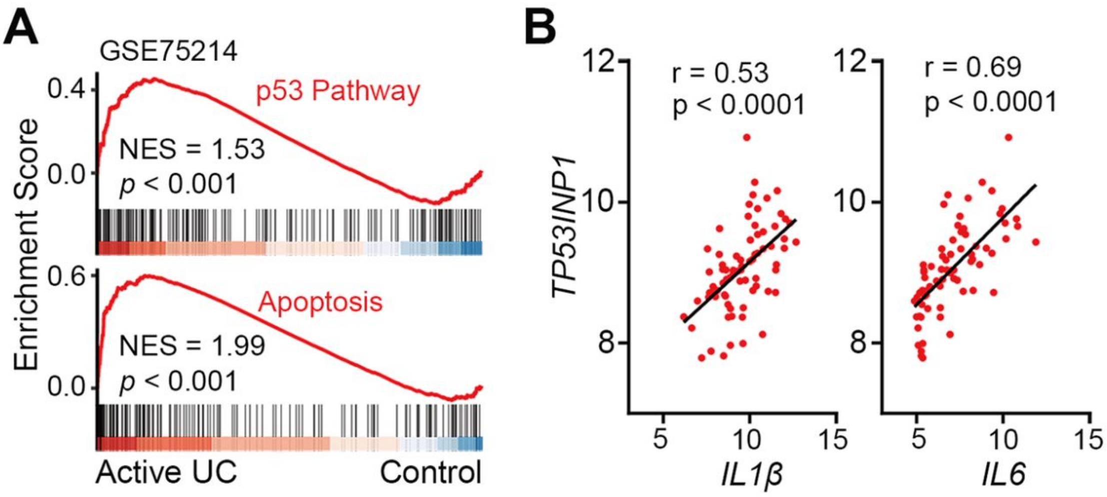

Figure S4. Activation of the p53 pathway and apoptosis in the colonic tissues from patients with active UC. (A) GSEA enrichment of the p53 pathway and apoptosis with increased genes in colonic tissues of patients with active UC from public dataset (GSE75214). (B) Pearson's correlation analysis showing the relationship between TP53INP1 level and IL-1β (left) level or IL-6 (right) levels in colonic tissues from patients with active UC.

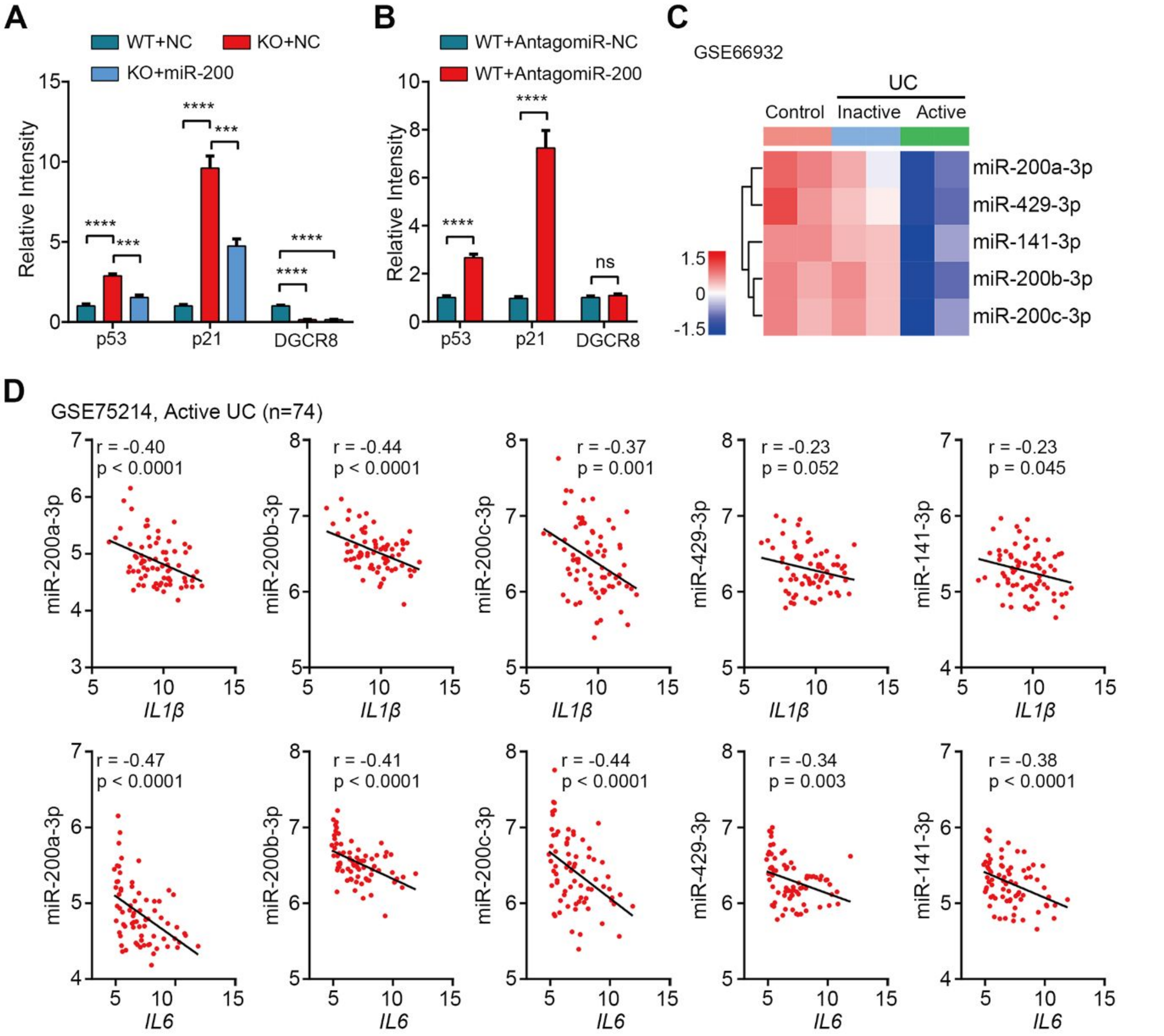

Figure S5. Downregulation of the miR-200 family in colonic tissues correlates with the pathogenesis of UC. (A,B) Quantification of western blot for p21, p53, DGCR8 in organoids (Figure 6H). (C) Heatmap showing the relative expression level of miR-200 family members in colonic tissues from healthy controls and patients with inactive UC or active UC (GSE66932). (D) Pearson's correlation analysis revealing the relationship between the level of miR-200 family members and pro-inflammatory cytokines interleukin IL-6 level or IL-1β level in colonic tissues from UC patients (GSE75214). *p* value was generated by unpaired two-tailed Student's *t*-test (A,B). ns, not significant, $p>0.05$; ***$p<0.001$; ****$p<0.0001$.****$p<0.0001$.

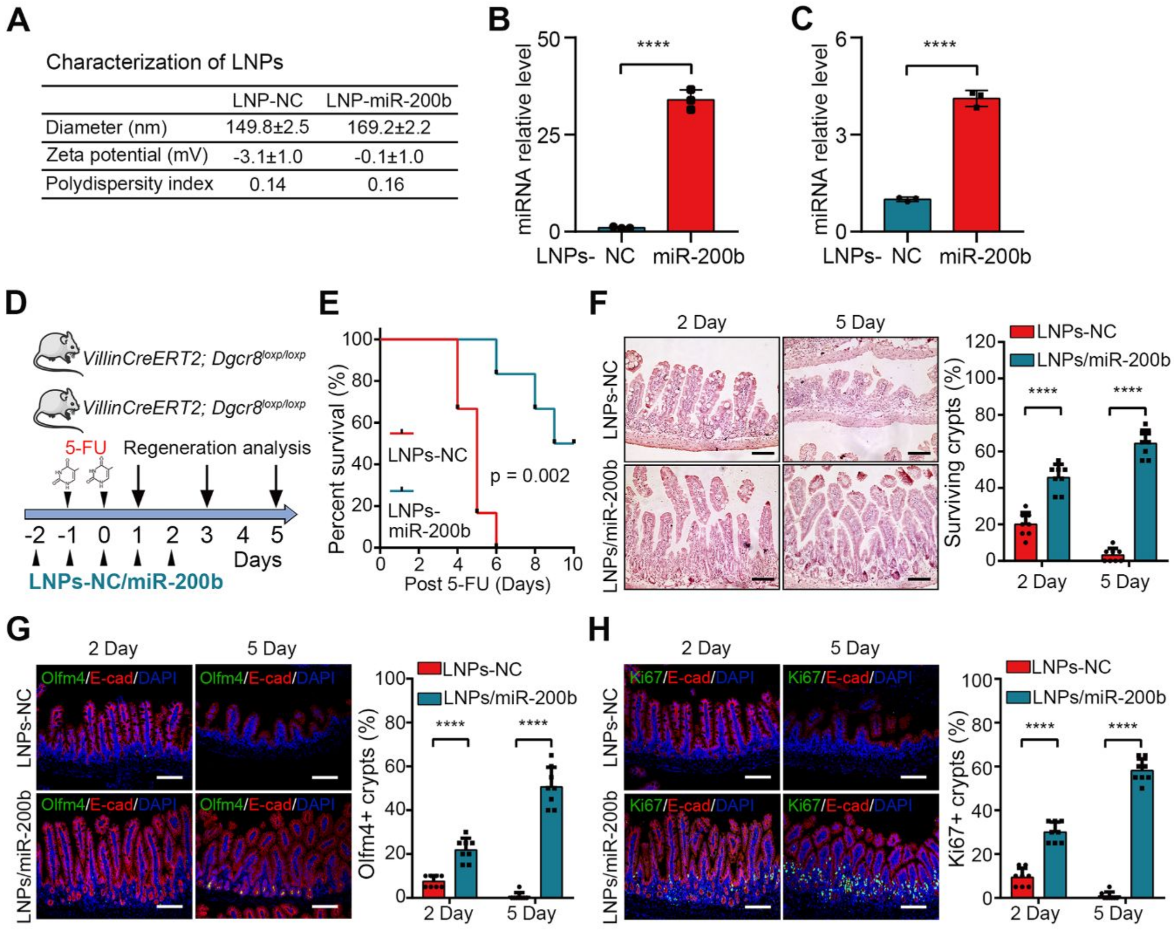

Figure S6. Oral delivery of miR-200 via lipid nanoparticles promotes epithelial regeneration of *Dgcr8*-deficient mice after 5-FU-induced injury. (A) Characterization of LNPs. (B,C) Relative levels of miR-200 in Caco-2 cells after incubation with LNPs-miR-200 and in intestinal epithelial cells of mice treated with oral gavage of LNPs-miR-200. (D) Schematic diagram showing 5-FU treatment in *Dgcr8* KO mice along with gavage of LNPs-NC/miR-200b. (E) The Kaplan-Meier survival curve of5-FU-treated *Dgcr8* KO mice after oral gavage of 200 μl LNPs-NC/miR-200b (miR-200b, 1.5 mg/kg by body weight) for 5 consecutive days. n = 6 biological replicates of each group. (F) Histological images of small intestines (left) and percentage of surviving crypts (right) from 5-FU-treated *Dgcr8* KO mice at indicated time points after oral gavage of LNPs-NC/miR-200b. (G,H) IF staining (left) and quantification (right) of Olfm4^{+} ISCs (H) and Ki67^{+} TA cells (I) in the intestinal epithelium from 5-FU-treated

Dgcr8 KO mice at indicated time points after oral gavage of LNPs-NC/miR-200b. Statistics data represent mean with SD (G-I) or mean with SEM (A). *p* values were generated by unpaired two-tailed Student's *t*-test (F-H) and one-sided log-rank test (D). ****$p<0.0001$. Scale bars: 20 μm (C), 50 μm (D), 100 μm (F-H).

Table S1. Primers for qRT-PCR of mRNAs.

Primer	Sequence (Forward)	Sequence (Reverse)
Gapdh	TGGCCTTCCGTGTTCCTAC	GAGTTGCTGTTGAAGTCGCA
Dgcr8	GGAGAGACAAGTGTACAGCCAATG	TCTGTGCAGGTATACAGGGACTCCA
Lgr5	TAAAGACGACGGCAACAGTG	GCCTTCAGGTCTTCCTCAAA
Olfm4	CAGCCACTTTCCAATTTCACTG	GCTGGACATACTCCTTCACCTTA
Ki67	ATCATTGACCGCTCCTTTAGGT	GCTCGCCTTGATGGTTCCT
Ascl2	GCCTGACCAAATGCCAAGTG	ATTTCCAAGTCCTGATGCTGC
Axin2	ATGAGTAGCGCCGTGTTAGTG	GGGCATAGGTTTGGTGGACT
Sox9	AGTACCCGCATCTGCACAAC	ACGAAGGGTCTCTTCTCGCT
Sox4	GACAGCGACAAGATTCCGTTC	GTTGCCCGACTTCACCTTC
Fzd2	CTTCACGGTCACCACCTATTTAG	CGCAATGTAGGCCACTGACA
Jun	ACTCGGACCTTCTCACGTC	GGTCGGTGTAGTGGTGATGT
Cdk6	GGCGTACCCACAGAAACCATA	AGGTAAGGGCCATCTGAAAACT
Cdca7	ATGTCATCAGTGTCGCCAGAAA	CCTCGCCATAGCGGTTTCG
Mcm5	TGAACTCAAGCGGCATTACAA	GGCTGTTTATGCAAGTGGTCA
Mcm6	GCTGTTCCTAGACTTCCTGGA	CAACCAGCGTGTTTCTCTCAG
Pcna	TTGCACGTATATGCCGAGACC	GGTGAACAGGCTCATTCATCTCT
Fas	GCGGGTTCGTGAAACTGATAA	GCAAAATGGGCCTCCTTGATA
Cdkn1a	CCTGGTGATGTCCGACCTG	CCATGAGCGCATCGCAATC
Trp53inp1	TCTCAGTGAGGCGAGTTGTGGA	ATCCACTGGGAAGGGCGAAAAC
Atg9b	GAGCAGGACTATGAACGGCTAG	GTCCAGGTTCTGGATGTGATGC

Table S2. Primers for reverse transcription of miRNAs.

Primer	Sequence (Forward)
RNU6B	CGCTTCACGAATTTGCGTGTCAT
miR-192-5p	GTCGTATCCAGTGCAGGGTCCGAGGTATTCGCACTGGATACGAC TGTCAA
let-7c-5p	GTCGTATCCAGTGCAGGGTCCGAGGTATTCGCACTGGATACGAC ATACAA
miR-429-3p	GTCGTATCCAGTGCAGGGTCCGAGGTATTCGCACTGGATACGAC CATTAC
miR-191-5p	GTCGTATCCAGTGCAGGGTCCGAGGTATTCGCACTGGATACGAC GCTTTT
miR-26a-5p	GTCGTATCCAGTGCAGGGTCCGAGGTATTCGCACTGGATACGAC TATCCT
let-7a-5p	GTCGTATCCAGTGCAGGGTCCGAGGTATTCGCACTGGATACGAC ATACAA
let-7f-5p	GTCGTATCCAGTGCAGGGTCCGAGGTATTCGCACTGGATACGAC ATACAA
let-7b-5p	GTCGTATCCAGTGCAGGGTCCGAGGTATTCGCACTGGATACGAC ACACAA
miR-200a-3p	GTCGTATCCAGTGCAGGGTCCGAGGTATTCGCACTGGATACGAC CGTTAC
miR-16-5p	GTCGTATCCAGTGCAGGGTCCGAGGTATTCGCACTGGATACGAC AATATT
let-7i-5p	GTCGTATCCAGTGCAGGGTCCGAGGTATTCGCACTGGATACGAC GCACAA
let-7g-5p	GTCGTATCCAGTGCAGGGTCCGAGGTATTCGCACTGGATACGAC GTACAA
miR-200c-3p	GTCGTATCCAGTGCAGGGTCCGAGGTATTCGCACTGGATACGAC CATTAC
let-7d-5p	GTCGTATCCAGTGCAGGGTCCGAGGTATTCGCACTGGATACGAC ATGCAA
miR-378a-3p	GTCGTATCCAGTGCAGGGTCCGAGGTATTCGCACTGGATACGAC TCTGAC
miR-92a-3p	GTCGTATCCAGTGCAGGGTCCGAGGTATTCGCACTGGATACGAC GCCGGG
miR-194-5p	GTCGTATCCAGTGCAGGGTCCGAGGTATTCGCACTGGATACGAC CATGGA
miR-375-3p	GTCGTATCCAGTGCAGGGTCCGAGGTATTCGCACTGGATACGAC GCGAGC
miR-23b-3p	GTCGTATCCAGTGCAGGGTCCGAGGTATTCGCACTGGATACGAC AATCCC
miR-21a-5p	GTCGTATCCAGTGCAGGGTCCGAGGTATTCGCACTGGATACGAC CATCAG
miR-181a-5p	GTCGTATCCAGTGCAGGGTCCGAGGTATTCGCACTGGATACGAC CCGACA
miR-34a-5p	GTCGTATCCAGTGCAGGGTCCGAGGTATTCGCACTGGATACGAC CCAGCT
miR-93-5p	GTCGTATCCAGTGCAGGGTCCGAGGTATTCGCACTGGATACGAC TGCACG

miR-200b-3p	GTCGTATCCAGTGCAGGGTCCGAGGTATTCGCACTGGATACGAC CATTAC
miR-10a-5p	GTCGTATCCAGTGCAGGGTCCGAGGTATTCGCACTGGATACGAC ATTCGG
miR-27b-3p	GTCGTATCCAGTGCAGGGTCCGAGGTATTCGCACTGGATACGAC GAACTT
miR-24-3p	GTCGTATCCAGTGCAGGGTCCGAGGTATTCGCACTGGATACGAC TCCTGC
miR-151-5p	GTCGTATCCAGTGCAGGGTCCGAGGTATTCGCACTGGATACGAC AGACTG
miR-30b-5p	GTCGTATCCAGTGCAGGGTCCGAGGTATTCGCACTGGATACGAC GAGTGT
miR-31-5p-	GTCGTATCCAGTGCAGGGTCCGAGGTATTCGCACTGGATACGAC TATGCC
miR-423-3p	GTCGTATCCAGTGCAGGGTCCGAGGTATTCGCACTGGATACGAC GGGGCC
miR-141-3p	GTCGTATCCAGTGCAGGGTCCGAGGTATTCGCACTGGATACGAC CTTTAC
miR-183-5p	GTCGTATCCAGTGCAGGGTCCGAGGTATTCGCACTGGATACGAC AATTCT
miR-182-5p	GTCGTATCCAGTGCAGGGTCCGAGGTATTCGCACTGGATACGAC AGTTCT
miR-652-3p	GTCGTATCCAGTGCAGGGTCCGAGGTATTCGCACTGGATACGAC AACCCT
miR-7a-5p	GTCGTATCCAGTGCAGGGTCCGAGGTATTCGCACTGGATACGAC AAAATC

Table S3. Primers for quantitative real-time PCR of miRNAs.

Primer	Sequence
Universal-reverse	CGCTTCACGAATTTGCGTGTCAT
RNU6B-forward	GCTTCGGCAGCACATATACTAAAAT
miR-192-5p-forward	CGCGCGCTGACCTATGAA
let-7c-5p-forward	GCGCGCGTGAGGTAGTAGG
miR-429-3p-forward	CGCGCGCGTAATACTGTCTG
miR-191-5p-forward	CGCGCAACGGAATCCC
miR-26a-5p-forward	GCGCGCGTTCAAGTAATCC
let-7a-5p-forward	GCGCGCGTGAGGTAGTAGG
let-7f-5p-forward	GCGCGCGTGAGGTAGTAGA
let-7b-5p-forward	GCGCGCGTGAGGTAGTAGG
miR-200a-3p-forward	GCGCGCGTAACACTGTCTG
miR-16-5p-forward	CGCGCGTAGCAGCACGTA
let-7i-5p-forward	GCGCGCGTGAGGTAGTAGT
let-7g-5p-forward	GCGCGCGTGAGGTAGTAGT
miR-200c-3p-forward	GCGCGTAATACTGCCGG
let-7d-5p-forward	GCGCGCGAGAGGTAGTAGG
miR-378a-3p-forward	CGCGCGACTGGACTTGGA
miR-92a-3p-forward	CGCGCGTATTGCACTTGT
miR-194-5p-forward	CGCGCGTGTAACAGCAAC
miR-375-3p-forward	GCGCGTTTGTTCGTTCG
miR-23b-3p-forward	GCGCGATCACATTGCCA
miR-21a-5p-forward	GCGCGCGTAGCTTATCAGA
miR-181a-5p-forward	GCGCGAACATTCAACGC
miR-34a-5p-forward	GCGCGTGGCAGTGTCTT
miR-93-5p-forward	GCGCGCAAAGTGCTGTT
miR-200b-3p-forward	CGCGCGTAATACTGCCTG
miR-10a-5p-forward	GCGCGCGTACCCTGTAGAT
miR-27b-3p-forward	GCGCGTTCACAGTGGCT
miR-24-3p-forward	GCGCGTGGCTCAGTTCA
miR-151-5p-forward	GCGCGTCGAGGAGCTCA
miR-30b-5p-forward	GCGCGCGTGTAAACATCCT
miR-31-5p-forward	GCGCGAGGCAAGATGCT
miR-423-3p-forward	GCGCGAGCTCGGTCTGA
miR-141-3p-forward	GCGCGCGTAACACTGTCTG
miR-183-5p-forward	GCGCGTATGGCACTGGT
miR-182-5p-forward	GCGCGTTTGGCAATGGT
miR-652-3p-forward	CGCGAATGGCGCCACT
miR-7a-5p-forward	GCGCGCGTGGAAGACTAGT

Table S4. Primers for miR-200b-3p binding sites in the dual luciferase reporter assay.

Primer	Sequence
miR-200b-Positive-forward	CTAGCCATCTTTACCAGACAGTGTTA
miR-200b-Positive-reverse	AGCTTAACACTGTCTGGTAAAGATGG
miR-200b-Negative-forward	CTAGTAACACTGTCTGGTAAAGATGG
miR-200b-Negative-reverse	AGCTCCATCTTTACCAGACAGTGTTA
miR-200b-p53-forward	CTAGTATCCAGCCAGTTGTTGGACCCTG
miR-200b-p53-reverse	AGCTCAGGGTCCAACAACTGGCTGGATA
miR-200b-p21-forward	CTAGTCCTTTCTCAGTGTTGAATACCG
miR-200b-p21-reverse	AGCTCGGTATTCAACACTGAGAAAGGA
miR-200b-p53 mut-forward	CTAGTATCAGGGTCCAACAACTGGCTGG
miR-200b-p53 mut-reverse	AGCTCCAGCCAGTTGTTGGACCCTGATA
miR-200b-p21 mut-forward	CTAGTCCCGGTATTCAACACTGAGAAA
miR-200b-p21 mut-reverse	AGCTTTTCTCAGTGTTGAATACCGGGA
